# Electrospray-Induced Mass Spectrometry Is Not Suitable for Determination of Peptidic Cu(II) Complexes

**DOI:** 10.1021/jasms.1c00206

**Published:** 2021-11-05

**Authors:** Dawid Płonka, Radosław Kotuniak, Katarzyna Dąbrowska, Wojciech Bal

**Affiliations:** Institute of Biochemistry and Biophysics, Polish Academy of Sciences Pawińskiego 5A, 02-106 Warsaw, Poland

## Abstract

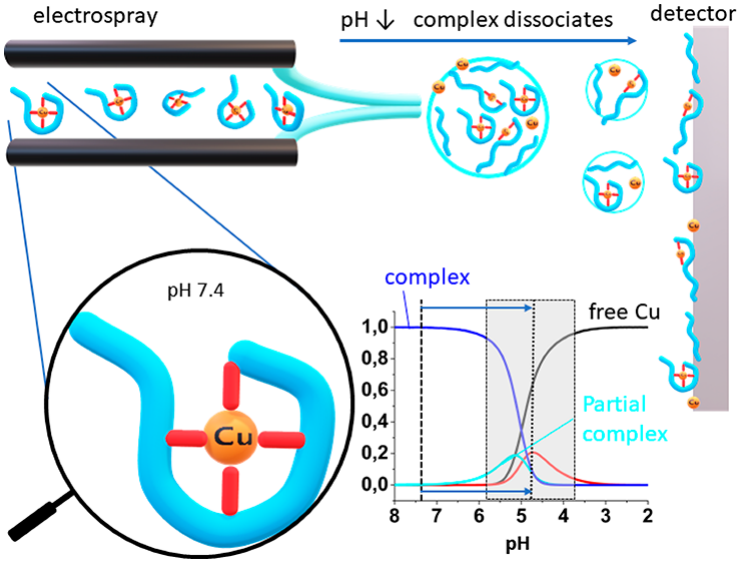

The toolset of mass spectrometry (MS) is still expanding, and the number of metal ion complexes researched this way is growing. The Cu(II) ion forms particularly strong peptide complexes of biological interest which are frequent objects of MS studies, but quantitative aspects of some reported results are at odds with those of experiments performed in solution. Cu(II) complexes are usually characterized by fast ligand exchange rates, despite their high affinity, and we speculated that such kinetic lability could be responsible for the observed discrepancies. In order to resolve this issue, we selected peptides belonging to the ATCUN family characterized with high and thoroughly determined Cu(II) binding constants and re-estimated them using two ESI-MS techniques: standard conditions in combination with serial dilution experiments and very mild conditions for competition experiments. The sample acidification, which accompanies the electrospray formation, was simulated with the pH–jump stopped-flow technique. Our results indicate that ESI-MS should not be used for quantitative studies of Cu(II)–peptide complexes because the electrospray formation process compromises the entropic contribution to the complex stability, yielding underestimations of complex stability constants.

## Introduction

**Figure 1:**
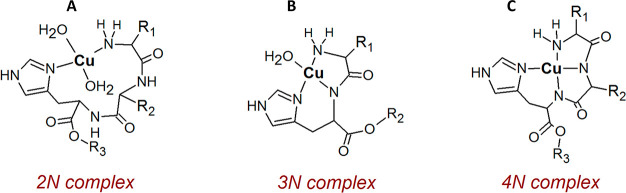
Structures of Cu(II) complexes at the N-terminal site of peptides having His in the 2^nd^ (B) or 3^rd^ position (A) and (C). R_(n)_ mark side chains and the remainder of the peptide chain.

Complexation to peptides is proposed to play important roles in Cu(II) physiology and toxicology. Cu(II) activates GHK, a wound healing factor^1,2^ and α-factor, a yeast pheromone,^3,4^ and is likely transported to neurons by neurokinin B.^5^ It also elicits toxicity and probably gets detoxified by some variants of Aβ peptides^6,7^ and protamine HP2^8−10^ and likely participates in the antifungal action of histatins, salivary antimicrobial peptides.^11^ A recent study indicated that more peptides with such properties remain to be identified in human proteome.^12^ Peptides have also been used extensively to model Cu(II) binding to its transport proteins, such as albumin and hCtr1 membrane transporter,^13−15^ and synaptic proteins, such as prions, APP, and α-synuclein.^16,17^ Two N-terminal sequence motifs, Xaa-His and Xaa-Zaa-His (where Xaa is any α-amino acid except of Cys, and Zaa is any α-amino acid except of Cys or Pro), provide the highest Cu(II) complex affinities by virtue of synergistic formation of chelate rings involving peptide nitrogen atoms (Figure 1).^18−20^ The logarithmic conditional stability constants at physiological pH 7.4, log ^C^*K*_*7.4*_, for Xaa-His complexes are in the range of 12.5–13, while those of Xaa-Zaa-His complexes range from 12.3 to ca. 15.^21,22^ The latter are also known as ATCUN or NTS complexes.^18^

Mass spectrometry (MS) in its many variants is one of the most versatile techniques of peptide and protein research, providing information on their composition, sequence, and post-translational modifications and via hyphenated techniques also on protein structure. It has also been widely used to determine affinity constants of molecular complexes, using a number of experimental approaches,^23−27^ including studies of metal complexes.^28^ A significant work was devoted to developing best conditions for quantitative determinations, including optimization of spray formation.^29,30^

Under certain conditions, MS can also be used to obtain quantitative information on metal ion complexes, e.g., Zn(II), Cu(I) or As(III).^31−34^ It has also been used to determine the binding constants for Cu(II) complexes.^34−36^

One issue to consider is the rate at which the charged molecular ions are generated with respect to parallel changes in their environment. During the electrospray formation the time from injection of the sample from the capillary to the droplet fission takes a few milliseconds.^37−39^ Many metal ion complexes remain intact in this time scale due to their sufficient inertness in ligand exchange reactions.^40^ However, as recently demonstrated by stopped-flow and freeze-quench techniques, the formation of Cu(II) complexes with ATCUN peptides Gly-Gly-His and Aβ_4–16_ proceeds via a reactive intermediate step whose lifetime is in the hundred milliseconds range.^20,41^ The Cu(II) ion in this species is partially coordinated to the amine and imidazole nitrogens only (Figure 1A) and is considered to be kinetically labile. Such lability may in turn affect the apparent binding constant determination by exposing partially folded/coordinated complexes to the gas phase. Moreover, alternative complex stoichiometries can occur. For example, Matsumoto et al. discovered Cu(II) complexes that emerged rapidly in the gas phase but were not present in solution.^42^

In addition, the presence of stable, e.g., “physiological”, conditions in the gas phase is very debatable. Protein and peptide structures are maintained through covalent bonds, hydrogen bonds, and hydrophobic interactions with a strong contribution from the solvent entropy.^43,44^ The pH and ionic strength cannot be defined anymore for the molecule after its transition to gas phase. Protonation states are altered^45^ and hydrophobic interactions get lost altogether, along with the evaporation of solvating water molecules.^46^ In contrast, electrostatic interactions, hydrogen bonds and van der Waals interactions are enhanced in the gas phase, prompting the loss of native structure.^46^ The MS measurement time scale is generally considered as too short for the protein or peptide unfolding, because it requires a concerted breaking of multiple noncovalent bonds.^47,48^ Some proteins may actually unfold in less than 1 ms, however.^49^ Metal binding to a peptide contributes only a few coordination bonds, typically two to six, and perhaps several weaker interactions in the second coordination sphere. The loss of even one of them due to gas phase conditions will have a profound effect on the complex stability. This is particularly important for peptidic Cu(II) complexes in which the entropic contribution to stability is very significant.^50,51^

Next, the equilibrium could be shifted because the pH of electrospray droplets might be different from that set in the sample prior to the analysis. Finding an appropriate buffer for ESI-MS studies is a difficult task.^52^ Most buffers that maintain physiological pH do not qualify because their ionic character suppresses the signal. Ammonium acetate is commonly used as a “buffer” for so-called native ESI-MS experiments due to volatility of neutral forms of its components, acetic acid and ammonia, which form upon the spray evaporation and do not contribute to ionic noise. However, as pointed out by Konermann, ammonium acetate is not really a buffer at pH 7.^52^ It has two buffering areas around the p*K* values of its components, at 4.75 ± 1 for acetate and 9.25 ± 1 for ammonia. Hence, the rising number of H^+^ ions produced by electric field in shrinking solvent droplets in the positive ion measurement mode rapidly decreases the droplet pH, down to the acetate buffering range of 4.75 ± 1. Under these circumstances there is no straightforward way of determining how exactly the pH has changed during the measurement. This poses a serious problem for quantitative analysis because the binding constants may change drastically with pH (see Table 1). The situation is further aggravated when mixtures of water and organic solvents are used, making the pH even more difficult to ascertain.^53^ The discrepant effects of droplet acidification and other effects mentioned above on the characterization of metal ion complexes have been noted, e.g., for relatively weak complexes of alkaline earth metal ions with EDTA,^54^ or lanthanide complexes with acetate,^55^ but also for very tightly bound species, e.g., the Bi^3+^ complex of transferrin.^56^

**Table 1:** Comparison of Conditional Cu(II) Binding Constants of the Studied Peptides at pH = 7.4 (^C^*K*_7.4_), Compared to These Constants Determined Previously by Other Methods and Published^a^

peptide sequence (name)	published log ^C^*K*_7.4_^b^	log *K*_d_ ± SD from serial dilution ESI-MS experiment in this study	log ratio log *K*_d_ (ESI-MS) – log ^C^*K*_7.4_ (published)	range of concentrations in this study (μM)
DTHFPI-NH_2_(hepc6)	14.7^c^	7.6 ± 0.7	–7.1	100–0.01
MNH-NH_2_	14.5^d^	6.3 ± 0.4	–8.2	100–2
FRHDSG (Aβ4–9)	14.2^e^	6.9 ± 0.9	–5.3	100–1
FRHDSGYEVHHQK-NH_2_ (Aβ4–16)	13.5^f^	7.0 ± 0.2	–6.5	100–0.5
MDH-NH_2_	13.1^d^	7.1 ± 0.6	–6.0	100–1
GGH	12.2^g^	6.0 ± 0.5	–6.2	100–10

a Standard deviation of ESI-MS experimental values are given in parentheses. Logarithmic values are given for better clarity. The published log ^C^*K*_7.4_ values were determined by potentiometry and corroborated by spectroscopic methods.

b The SD values were considerably less than 0.1 log unit in all cases.

c Reference (63).

d Reference (64).

e Reference (65).

f Reference (6).

g Reference (21).

Other available buffers offer little alternative. Ammonium bicarbonate has p*K*_a_ = 6.4 but is unstable and prone to CO_2_ evolution, while common biologically friendly buffers, like Tris or HEPES, contain nonvolatile cations and anions, resulting in a poor MS data quality.

Furthermore, redox-prone metal complexes, including the Cu(II) species, may undergo reduction in the gas phase. This effect is independent of the method of ionization, as such behavior was reported in both electrospray and plasma desorption studies.^57,58^ It is difficult to monitor and can influence quantitative studies, as the copper oxidation state is crucial for the complex formation and stability. For example, the ESI-MS experiments on the Cu(II)–GHK peptide complex yielded a mixture of Cu(II) and Cu(I) species in a proportion depending on the electrostatic potential in the ion source.^59^ Tsybizova et al. listed three possible mechanisms for Cu(II) reduction: reduction on the capillary walls, desolvation with electron transfer and reduction while still in the solution.^60^ Reduction was more likely to occur when the Cu(II) ion was coordinated by no more than two atoms.

The unpredictability of issues with reduction and pH shift is aggravated by an uncertainty of the measurement time scale which depends on the droplet size. As a general rule, the bigger the droplet is, the longer time it takes to dissipate, extending the opportunity for unwanted processes to occur.^61^

**Figure 2:**
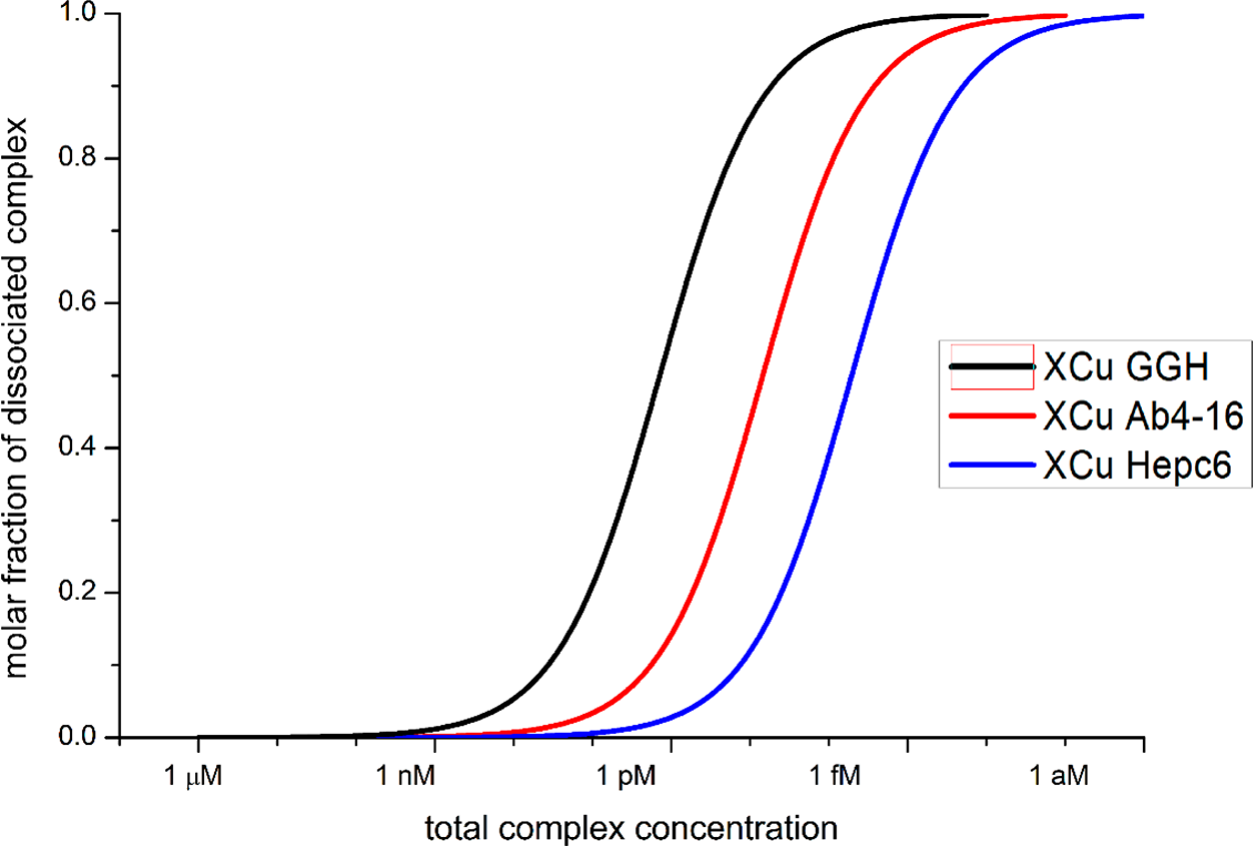
Theoretical dissociation curves of Cu(II)/peptide 1:1 complexes at pH 7.4.

In our laboratory practice, we frequently recorded ESI-MS spectra of Cu(II) complexes of peptides. Nearly always strong signals of unbound peptide were observed, although they were not present in equilibrium (see Figure 2 for examples). This strongly indicated that complex dissociation occurred in the mass spectrometer, yielding artifacts. On the other hand, ESI-MS is often treated, e.g. by less experienced researchers as if it faithfully reflected the solution equilibria. The goal of our study was therefore to systematically explore the issue of a systematic error in attempts to study Cu(II)/peptide complexes quantitatively by ESI-MS. In order to achieve it, we performed ESI-MS titration experiments on six high-affinity Cu(II)/peptide systems which we previously characterized in a comprehensive fashion.

We started with serial dilution experiments on a standard ESI-MS instrument in order to obtain apparent affinity constants serving for quantitative illustration of the scale of the systematic error generated by ESI-MS. This was followed by pH–jump kinetic experiments aimed to model events occurring in the evaporating spray droplets and by additional competition-based affinity studies performed on an ESI-MS instrument dedicated for studies of noncovalent interactions. All these experiments yielded a clear and unequivocal view that ESI-MS should not be used for quantitation of Cu(II)/peptide complexes.

## Experimental Section

l-Histidine and ammonium acetate were purchased from Sigma. All peptides, except for GGH purchased from Sigma, were synthesized in-house with standard Fmoc solid phase synthesis, as described before.^62^ Crude synthesis products were purified with RP-HPLC on ACE C18-300 column 250 × 8 mm with a rising gradient of acetonitrile in water with 0.1% TFA.

ESI-MS spectra were recorded on a Premier ESI-QToF spectrometer (Waters). All measurements were performed in the positive ion mode. The source temperature 80 °C was used for a complete desolvation of the peptide ions. The cone voltage was 10 V for shorter peptides and 30 V for Aβ_4–16_. The transmission of the ions was optimized on the quadrupole for the required mass range (*m*/*z* 200 to 1000 for shorter peptides, *m*/*z* 300 to 1500 for Aβ_4–16_). Mass spectra were accumulated over 2 to 3 min to improve the signal-to-noise ratio. The sample flow was 20 μL/min. The 100 μM Cu(II)-peptide solutions in 20 mM ammonium acetate at pH 7.4 were used for serial dilutions of the whole complex with the same ammonium acetate. For quantitation, all peaks corresponding to each charged state were integrated for each peptide. Ratios of free peptide to Cu(II)/peptide complex were extracted from the integration of MS intensities with the assumption of similar ionization efficacy. For each dilution the *K*_d_ was calculated according to eq 1, followed by the averaging as required (see the Results for details).
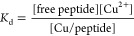
1The free Cu^2+^ ions could not be detected directly, due to low *m*/*z* and instead were calculated from the mass balance (free Cu^2+^ assumed to be equal to free peptide).

Isotopic distributions were calculated using built-in MassLynx Isotope model software (Waters).

Competition experiments with histidine were carried out on Q Exactive UHMR Hybrid Quadrupole-Orbitrap mass spectrometer (ThermoFisher Scientific). Samples were prepared by dissolving peptides in 50 mM ammonium acetate pH 7.4 in 100 nM concentration. To each sample a mixture of 100 nM CuCl_2_ and differing concentrations of histidine were added. Measurements started with an 1 h delay to allow for reaching equilibrium. Samples were introduced into the mass spectrometer with a syringe pump using 10 μL/min flow rate, by electrospray ionization using positive mode in HESI source. MS measurements were conducted under the following settings: desolvation voltage: 20 V, capillary temperature 320 °C, detector *m*/*z* optimization: low *m*/*z*; ion transfer optimization to low *m*/*z*. The RF applied throughout the instrument were set to 150 Vp-p for injection flatapole, 300 Vp-p for bent flatapole, 250 for transfer multipole and HCD cell, and 2300 for C-trap. The ions transfer optics was to 5 V for injection flatapole, 4 V for intel flatapole, 2 V for bent flatapole and 0 V for transfer multipole. Integration of the resulting peaks was achieved with built-in software FreeStyle 1.4 (Thermo Scientific).

The stopped-flow SFM-300 (BioLogic) instrument was used to measure the rates of dissociation of Cu(II)–GGH and Cu(II)–hepc6 complexes upon acidification. The kinetic runs were observed with a diode-array detector (TIDAS S 500 K, J&M Analitik AG), with the spectra recorded in the 400–900 nm wavelength range at 1.5 ms intervals. Reactions were performed in a 1 cm path length cuvette at 25 °C. The dead time of the instrument was 2 ms at total flow rate 15 mL/min.

The pH–jump experiments were carried out for solutions of 2 mM peptide and 1.8 mM CuCl_2_ in 50 mM ammonium acetate at pH = 7.4 mixed with equal volumes of 45 mM, 0.25 M, or 2.5 M acetic acid, which resulted in sample acidification to pH 5, 4, or 3, respectively. The dilution during the mixing yielded final cuvette concentrations of 1 mM peptide, 0.9 mM Cu(II), and 22.5 mM, 125 mM and 1.25 M acetic acid, respectively. The solutions were freshly prepared and degassed before each series of reactions. The final pH value was measured in the samples collected after each experiment. To show the differences in absorbance signal before the first recorded time point, the dilution of the Cu(II)–peptide complex was measured as a control (shown as *t* = 0 s). In all cases, the peptides were in a slight excess over Cu(II) to avoid Cu(OH)_2_ precipitation.

## Results

### ESI-MS Attempts at Determining ^C^K

To test the pseudophysiological ESI-MS conditions for the binding constant determination we employed two different mass spectrometers and two different techniques. Serial dilution studies of Cu–peptide complexes were performed on a standard ESI-MS instrument, while histidine competition experiments were performed on a sensitive instrument dedicated for studies of noncovalent complexes.

In the first approach, we performed measurements on several synthetic peptides with well-established Cu(II) binding properties. Equimolar Cu(II) peptide mixtures (100 μM) were dissolved in 20 mM ammonium acetate at pH 7.4. The dissociation of complexes was monitored by serial dilutions of both reagents. Table 1 provides the *K*_d_ values obtained according to eq 1 for each data point and averaged. These values were compared to the literature ^C^*K*_7.4_ values.^6,21,63−65^ Very large discrepancies were observed, ranging from five to eight log units.

The ESI-MS spectra are presented in Figures S1–S6. As seen in these figures, substantial amounts of unbound peptides were observed in all cases at all tested concentrations. For example, ca. 20% free peptide was detected throughout the dilution experiment for hepc6. This degree of Cu(hepc6) complex dissociation at pH 7.4 should not occur above a complex concentration of 9 fM (Figure 2).^63^ Further experiments were undertaken to explain the sources of this discrepancy.

For the peptides forming the weakest (GGH) and the strongest complexes (hepc6), different Cu(II)/peptide ratios were analyzed (Figure S7 and S8, respectively). The increasing Cu(II) amounts should eventually saturate the peptide, eliminating the apopeptide signals. This did not happen, even at the highest Cu(II) excess that could be achieved due to solubility, more than hundred-fold for GGH (Figure S7). As presented in Figure S9, increasing the Cu/peptide ratio lowered the apparent *K*_d_ value, contrary to what should be expected according to eq 1.

We also analyzed the spectra for the signs of Cu(II) reduction to Cu(I) as a possible source of *K*_d_ value deviation. This effect would manifest itself qualitatively in the ESI-MS spectra as a *m*/*z* shift of +1 for the molecular ions containing Cu(I), because the positive charge decrease on the copper ion would have to be compensated by an additional H^+^ ion attached elsewhere. This, in turn, would affect the peak proportions in the ionic manifold. This effect was analyzed by comparing the peak maximum intensities with respect to the monoisotopic (highest) peak. Indeed, very slight increases of the second and fourth peaks were observed in most peptides, consistent with the +1 mass shift, but the effect was small, not exceeding a few percent of the monoisotopic peak (Figure S10). Thus, some Cu(II) reduction to Cu(I) could have occurred in our experiments, but its contribution to *K*_d_ was minimal to negligible.

### Competition Experiments with Histidine

**Figure 3:**
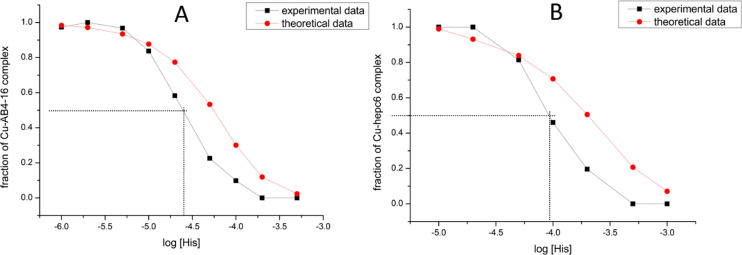
Experimental and theoretical binding isotherms for Cu(II)/Aβ4-16 (A) and Cu(II)/hepc6 (B) 100 nM 1:1 complexes titrated with histidine as a weak competitor. Dotted lines mark concentration at which 50% of the peptide is occupied with Cu(II) ion.

Having demonstrated that serial dilution experiments on a standard ESI-MS instrument inadvertently yield artifacts, we checked whether competition experiments could provide a better result. For that we used a state-of-the-art Q-Exactive Ultra-High Mass Range mass spectrometer designed specially to preserve native noncovalent bonds. The initial experiment in 50 mM ammonium acetate, pH 7.4, proved that no artifactual complex dissociation occurred for the CuAβ4–16 complex at the 100 pM concentration, near the instrument’s detection limit (Figure S11). According to Figure 2, this dilution was still 2 orders of magnitude away from the range at which serial dilution could be applied but gave promise for competition experiments. In this approach, 100 nM peptides Aβ4–16 and hepc6 mixed with 100 nM Cu(II) were titrated with histidine, which served as a weak competitor. With full knowledge of the protonation states and stability constants for each complex species in solution (Tables S1 and S2) we were able to calculate the theoretical binding isotherms indicating a feasibility of such approach. The experimental curves were unfortunately different (Figure 3).

Calculating binding constants from competition experiments where the competitor forms both 1:1 and 2:1 complexes is not an easy task in general, and competition may produce errors that are difficult to account for,^66^ but we were able to circumvent the calculation problem by extracting the histidine concentration at which 50% of peptides were bound to Cu(II) from Figure 3. These values were 24.9 μM His for Aβ4–16 and 93.3 μM His for hepc6. With these values and the knowledge of all protonation constants of histidine and stability constants for Cu(II)/histidine complexes^67^ we could calculate the binding constants using the competitivity index (CI) approach.^68,69^ The CI for a binary metal/ligand system is defined as the logarithm of the conditional stability constant of MZ (the metal complex of a theoretical molecule Z), such that Σ_*ijk*_([M_*i*_H_*j*_L_*k*_]) = [MZ], at given overall component concentrations, where M is a metal ion, H is hydrogen, and L is the metal binding ligand. In other words, the apparent stability constant of MZ, in units of M^–1^, reflects the overall metal ion binding ability of all other molecules in equilibrium. CI is equivalent to log ^C^*K*_7.4_ when there is only one complex species under given conditions but has a broader relevance if more than one species coexist in solution. In such case CI represents the metal binding properties of the whole ensemble of complex species. In the studied system L is histidine and Z is the peptide. The data obtained through these calculations are gathered in Table 2, and raw spectra are shown in Figures S12 and S13.

**Table 2:** Comparison of Conditional Cu(II) Binding Constants Obtained with Competition Experiments with Histidine of the Studied Peptides at pH = 7.4 (^C^*K*_7.4_), Compared to These Constants Determined Previously by Other Methods and Published^a^

peptide sequence (name)	published log ^C^*K*_7.4_	log *K*_d_ from His competition ESI-MS experiments in this study	log ratio log *K*_d_ (ESI-MS) - log ^C^*K*_7.4_ (published)	range of concentrations of histidine in this study (μM)
DTHFPI-NH_2_ (hepc6)	14.66^b^	13.98	–0.68	1000-10
FRHDSGYEVHHQK-NH_2_ (Aβ4–16)	13.53^c^	12.84	–0.69	500-1

a Logarithmic values are given for better clarity. The published log ^C^*K*_7.4_ values were determined by potentiometry and corroborated by spectroscopic methods.

b Reference (63).

c Reference (6).

### Modeling the pH Drop in Electrospray Droplets Using pH–jump Stopped Flow

**Figure 4:**
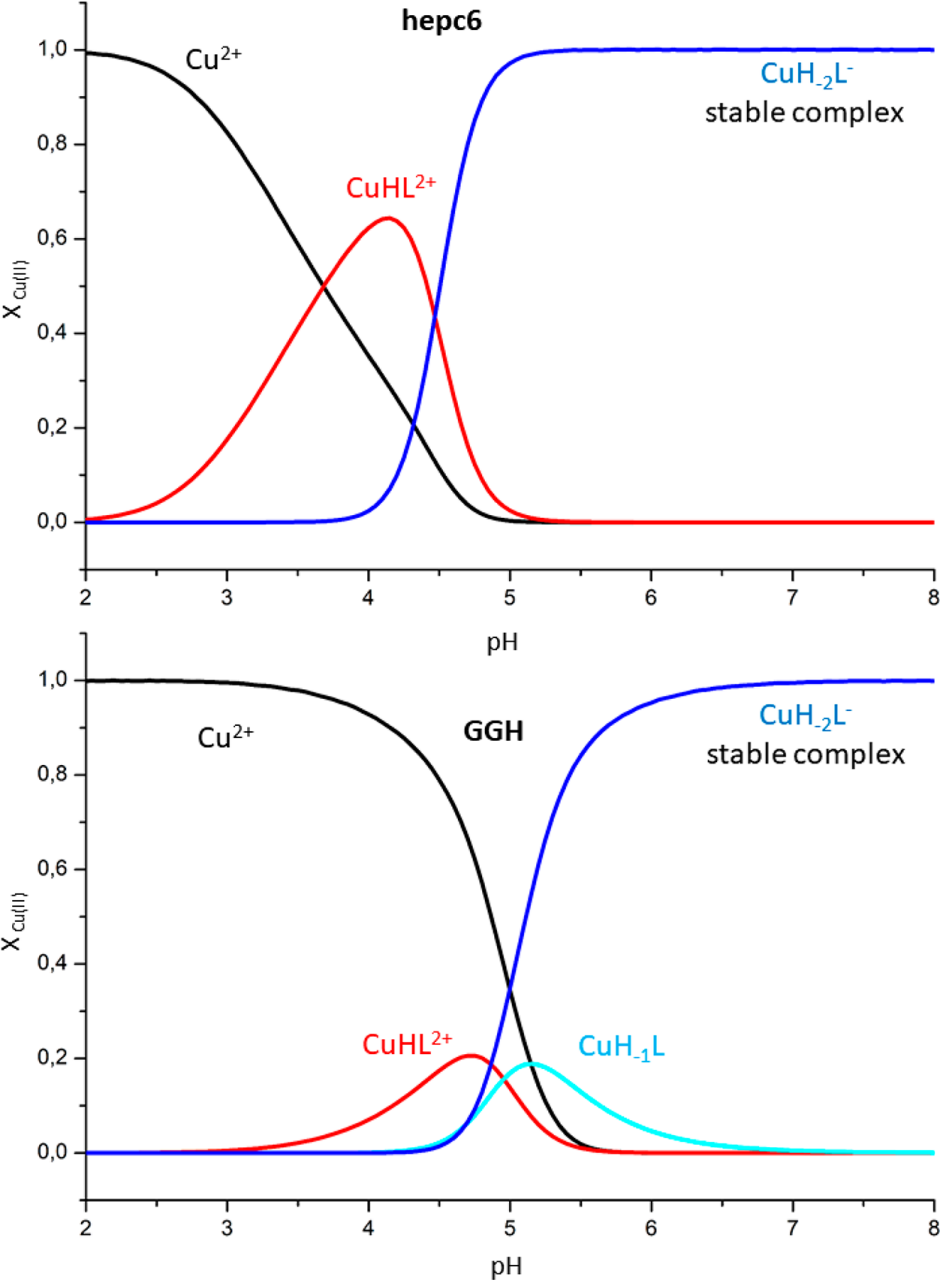
pH-dependent species distribution of Cu(II) complexes of hepc6 (top) and GGH (bottom) calculated for 1 mM peptides and 0.8 mM Cu(II) based on potentiometric data from ref (65) and ref (21), respectively.

The formation of Cu(II)–peptide complexes in general and ATCUN complexes in particular is very strongly pH-dependent.^18^ This is illustrated in Figure 4 for hepc6 and GGH and in Figure S14 for other studied peptides. These diagrams indicate that the significant coexistence of Cu(II)-bound and unbound peptides occurs typically in the pH range between 3 and 5. The presence of unbound peptides in all serial dilution ESI-MS experiments could thus be a simple consequence of acidification of sample droplets containing ammonium acetate during their evolution in electrospray.^52^

**Figure 5:**
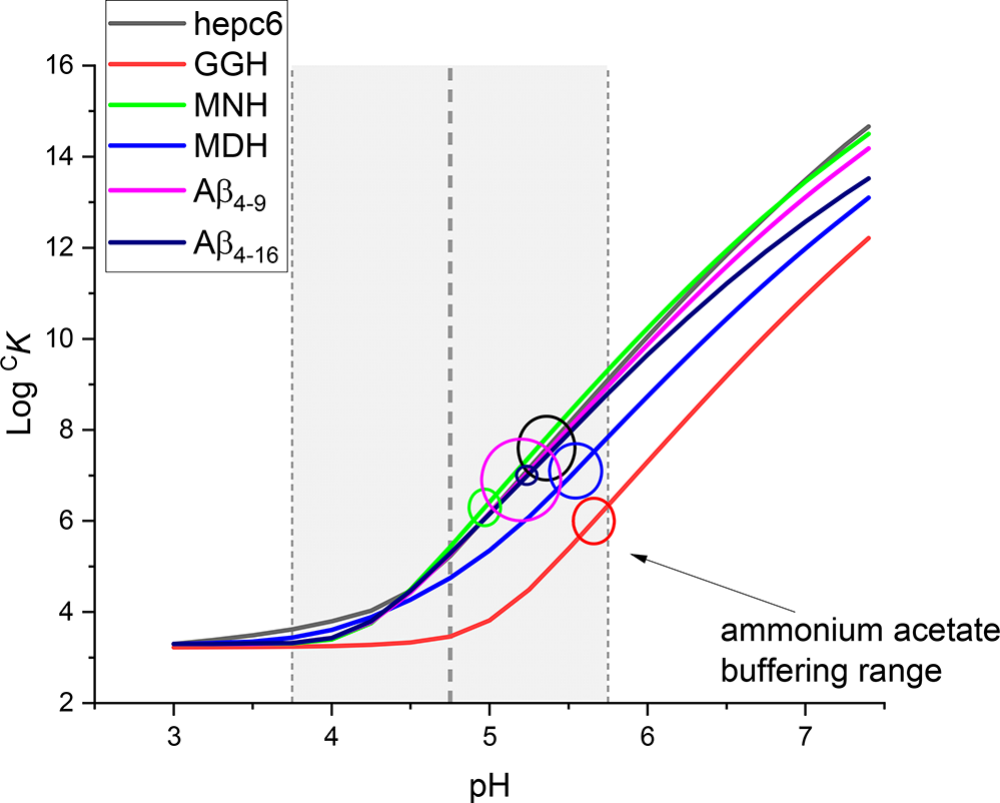
Prediction of pH values in electrospray stipulated by the literature log ^C^*K* values compared with the values calculated from serial dilution ESI-MS (data from Table 1). Solid curves represent the pH dependence of literature log ^C^*K* values for individual peptides. Circles mark the ESI-MS-derived log *K*_d_ values ± SD placed along these curves to indicate the pH of spray droplets expected if acidification were the sole source of the observed *K*_d_ decrease. Gray area marks the buffering zone of ammonium acetate (pH 4.75 ± 1).

Figure 5 provides the correlation diagram indicating what pH values in electrospray droplets would explain the apparent loss of Cu(II) affinity if droplet acidification were the only source of the observed log *K*_d_ decrease in serial dilution experiments. Although all values are within the buffering range of acetate, their spread is rather random and covers about one pH unit, despite using the same experimental conditions in all respective experiments. Therefore, the acidification, while plausible, is not the only source of the studied effect and the actual *K*_d_ values cannot be simply recovered from ESI-MS data by adjusting them for a hypothetical electrospray pH value.

**Figure 6:**
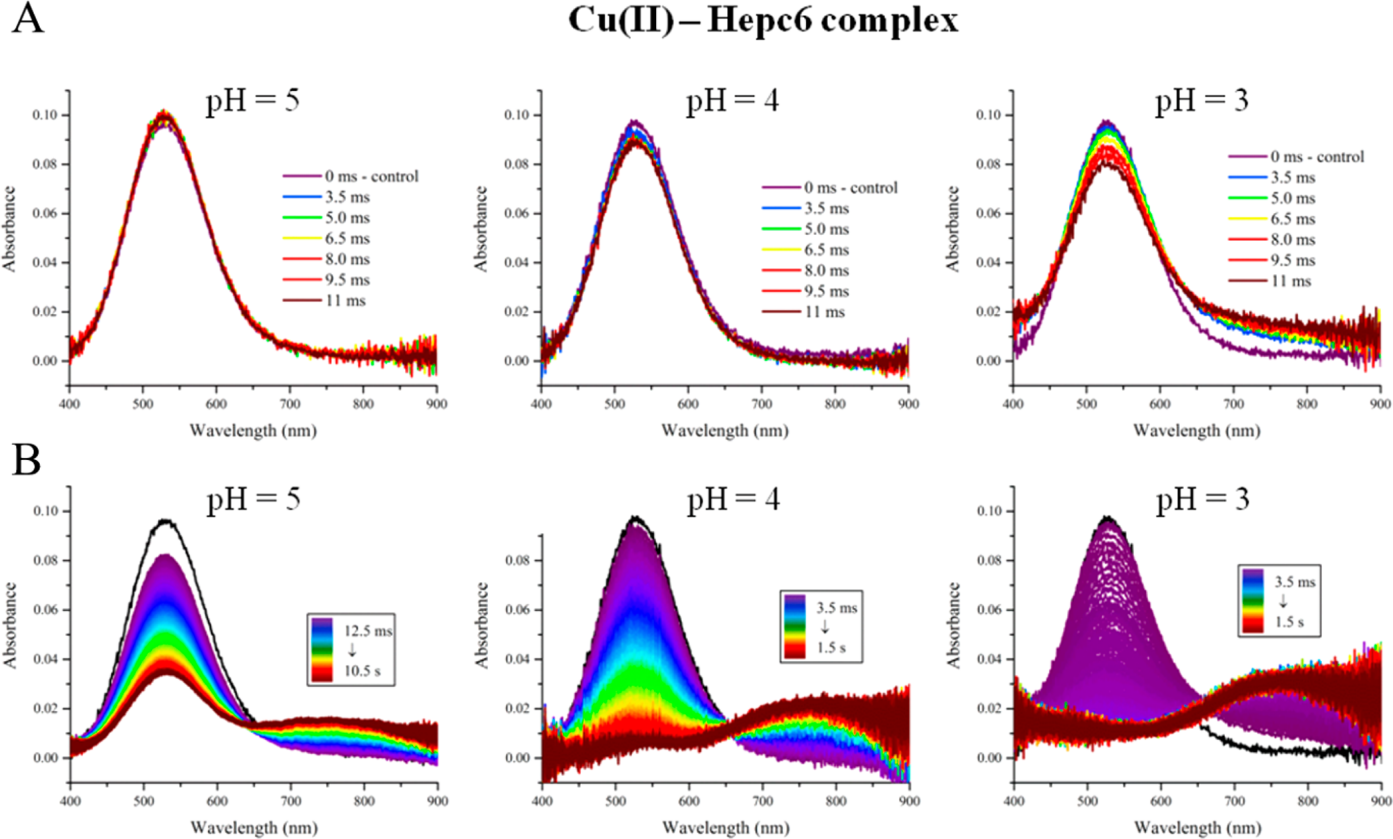
Stopped-flow spectra of Cu(II)-hepc6 in 20 mM ammonium acetate initially at pH 7.4, subjected to rapid pH decrease by mixing with concentrated acetic acid. Color-coded spectra were recorded every 1.5 ms with the instrument dead time of 2 ms. (A) First 11 ms of the reaction and (B) all spectra recorded until equilibrium was reached. The band at 525 nm represents the fully formed (4N) ATCUN complex, stable at pH 7.4, and the band at 750 nm represents the intermediate (2N) complex.^20^

Cu(II) complexes are usually considered to equilibrate rapidly, but our recent study demonstrated that Cu(II) complexes of ATCUN peptides are formed in a stepwise manner, with the lifetime of most stable intermediate species around several hundred milliseconds.^20^ A typical time of electrospray formation is ≤10 ms.^37,38^ These facts prompted us to investigate whether the complexes had enough time to reach the new pH-dependent equilibrium within the time of droplet evolution preceding the gas phase transition. To answer this question, we performed stopped-flow pH–jump experiments. The samples containing Cu(II)–hepc6 and Cu(II)–GGH complexes in ammonium acetate at pH 7.4 were mixed with acetic acid solutions of concentrations adjusted to reach pH 5, 4, or 3 after the sample mixing. The evolution of the systems was monitored using the visible absorption spectra, as shown in Figure 6 for Cu(II)–hepc6. The data for Cu(II)–GGH are provided in Figure S15.

**Figure 7:**
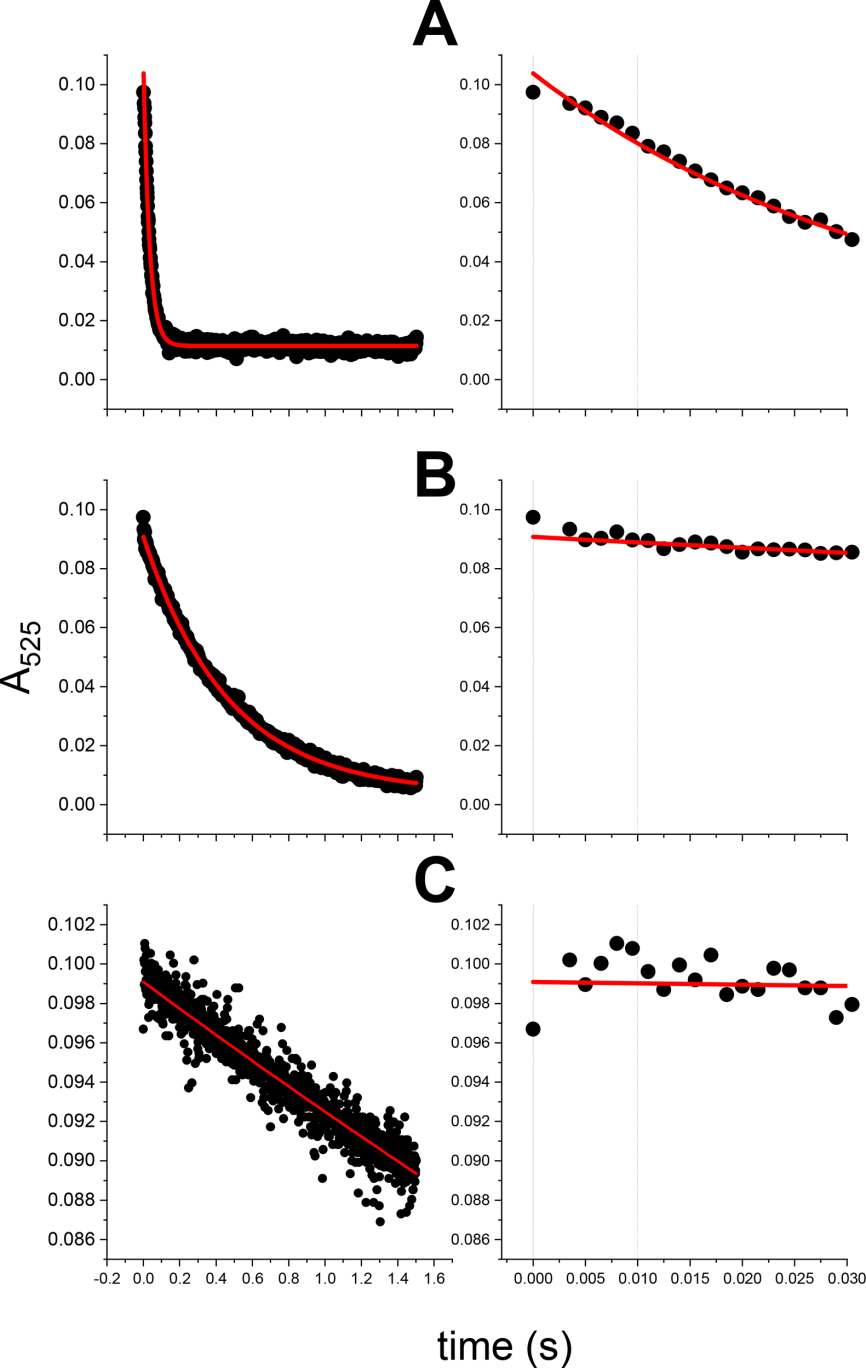
Kinetic traces at 525 nm for pH–jump experiments on Cu(II)-hepc6 presented in Figure 6 for pH 3 (A), 4 (B), and 5 (C). Left: full traces recorded over 1.5 s and 1st order kinetic fits (red lines). Right: the first 30 ms of traces. Vertical lines mark the 0 ms (reaction start) and the 10 ms time points.

The traces at 525 nm, corresponding to the decomposition of the 4N ATCUN complex, are presented in Figure 7 for Cu(II)–hepc6 and in Figure S16 for Cu(II)–GGH. The extent of dissociation of 4N complexes after 10 ms at pH 5, 4, and 3 was measured by following the decay of their absorption peaks at 525 nm. Due to significant noise of the absorption signal the averaged signal intensities at 0 and 10 ms were obtained by fitting a first order kinetic function to the absorption data. The overall fits and expanded regions of interest (0 to 30 ms) are presented for clarity. As seen in these figures, the inertness of ATCUN complexes to acid-catalyzed dissociation in the 10 ms time window is significant (except for Cu(II)–GGH at pH 3) and must be taken into account. Table S3 presents the results of a simulation of relative abundances of apopeptides and Cu(II) complexes that should occur if the ESI-MS signals depended only on droplet acidification and the resulting complex dissociation after 10 ms. This analysis indicates that the abundance of hepc6 apopeptide in ESI-MS spectra is consistent with pH the droplet drop to slightly above 3, while the abundance of GGH apopeptide appears to correspond with the final droplet pH clearly above 5. Both cannot be true for experiments performed under identical conditions. Hence, acidification and complex decay kinetics do not explain the pattern of free and complexed peptides observed in ESI-MS.

**Figure 8:**
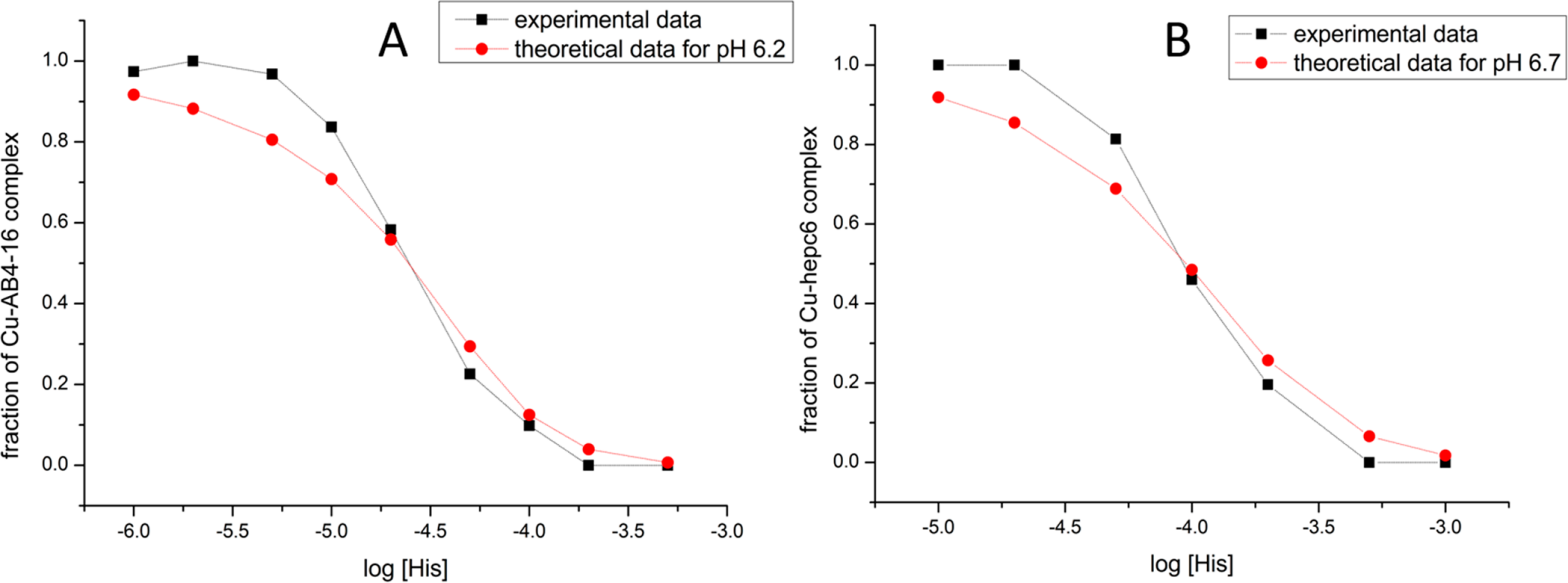
Experimental and theoretical binding isotherms of Cu/Aβ4-16 (A) and Cu/hepc6 (B) 1:1 complexes titrated with histidine as weak competitor.

The application of the above methodology to decipher the mechanism responsible for the deviation in competition experiments is not possible because of the overlap of the pH and desolvation effects exerted simultaneously on the peptide and histidine complexes. The droplet acidification and/or complex unfolding processes are less significant in the mild desolvation conditions of the respective instrument, as evidenced by the control experiment. The theoretical speciation for Cu(Aβ4–16) at the 100 pM concentration suggests that the pH drop may be actually very low or none (Figure S11). In accord, the attempt to assign a pH drop to competition experiments proved futile, as presented in Figure 8. Therefore, the systematic error embedded in these experiments is likely due to complex desolvation phenomena, as discussed below.

## Discussion

The experimental results presented in Figure 3 and Figures S1–S6 and S12–S13 showed that the proportions of free ATCUN peptides and their complexes in ammonium acetate solutions at pH 7.4, obtained by ESI-MS, do not match the expectations based on ^C^*K*_7.4_ values obtained by other techniques (Tables 1 and 2). This cannot be assigned to a nonproportionate efficacy of ionization of respective species, a well-known phenomenon in ESI-MS,^30^ because the discrepancy is far too high.

In the case of histidine competition experiments, a disproportional ionization would shift the binding curve but should not yield a steeper transition from free to complexed peptide. Doing so would indicate that ionization efficiency difference reverses in changing concentrations of the competitor. Interestingly, at low histidine concentrations the apparent Cu(II) binding by the peptides is slightly enhanced beyond the expected value. This could be caused by the fact that during shrinking of the droplet, when concentrations rise, His escapes the droplets earlier. This would shift the equilibrium in favor of the peptide. No such effect is observed, however, at higher histidine concentrations.

In the case of serial dilutions, the artifactual surplus of free peptides was not alleviated even at a high Cu^2+^ excess (Figures S7–S9). The analysis of relative peak intensities excluded the Cu(II) reduction to Cu(I) as a significant source of this discrepancy (Figure S10). The significant amounts of apopeptides in the spectra resembled the Cu^2+^/peptide equilibria under the acidic conditions (Figures 4 and S14), which appeared to correlate with the noted acidification of ammonium acetate solutions upon the electrospray droplet formation.^52^ Quantitative analysis demonstrated, however, that the apparent pH values derived from ESI-MS data are spread randomly over nearly one pH unit (5.0–5.7, Figure 5), effectively precluding a quantitative use of such data. Additionally, this apparent pH shift was less than expected for droplet acidification according to Konermann (around 4.75).^52^ Looking for a reason, we turned to pH–jump kinetic experiments, guided by our recent discovery of intermediate steps in the process of ATCUN complex formation, which could overlap with the lifetime of electrospray droplets.^20,41^ These experiments, presented in Figures 6, 7, S15, and S16, revealed that the studied complexes are far too inert to undergo a significant decomposition within the 10 ms time window facing the relevant extent of acidification. Therefore, the main reason for the complex dissociation was in this case an inadequate ESI source or its parameters. Tuning of voltage, gas flow, or injection flow did not yield considerable success. However, changing the spectrometer to the one dedicated to intact noncovalent bonds yielded much more accurate results. The apparent binding constants were much closer to expected values derived from solution studies but were still significantly underestimated.

We propose that the clue for the observed effect is provided by thermodynamics of Cu(II) complex formation. The ITC investigation of DAHK, an ATCUN peptide model of human serum albumin, demonstrated that the Cu(DAHK) complex at neutral pH is stabilized solely by entropic contribution.^50^ At this pH the enthalpic contributions such as Coulombic attraction are canceled out by the cost of deprotonations of peptide nitrogens some 8 orders of magnitude below its *K*_a_.^70^ The main contributor to complex stability is believed to be a release of water molecules from hydration shells of Cu(II) and the peptide, as well as deprotonation. Stable 4-nitrogen–Cu(II) complex leaves much less peptide and metal exposed to the solvent; thus, the entropic factor comes from the combination of folding and desolvation. A similar feature was observed for Cu(II) binding to metalloproteins.^51,71^ Therefore, in accordance with the data presented above, the removal of water from the second coordination sphere of a Cu(II)–ATCUN complex will likely lead to the equilibrium shift and possible subsequent complex decomposition upon entry of the molecules into the gas phase. It was previously established that thermodynamic changes occur during transition to the gas phase and entropically favored forces become destabilized.^28^ Additionally, acid-dissociated Cu^2+^ ions may recombine with the peptide upon its transition to the gas phase, additionally confounding the observations.^72^ From this perspective, one can state that neither ESI-MS nor other mass spectrometry techniques should be applied in quantitative studies of Cu(II)–peptide complexes. If such experiments are performed, the resulting binding constant will always be lower than the real one, but to an extent impossible to predict without independent data obtained by a different method. Therefore, mass spectrometry data for Cu(II)/peptide binding constants should be regarded as “greater than *x*”, always with an assumption that the in-solution binding constant is higher than that measured. On the other hand, enthalpic contributions are significant or decisive for the stability of other metal chelates in water,^73,74^ and hence, metal complex formation can be studied by MS upon maintaining the utmost scrutiny and validating the results by independent techniques whenever possible.

## Supporting Information Available

Serial dilution data (Figures S1–S6), Cu^2+^ titrations (Figures S7 and S8) and derived apparent *K*_d_ (Figure S9), isotopic distributions for detection of Cu^2+^ reduction (Figure S10), calculated pH distribution of Cu(II)Aβ4-16 (Figure S11), raw spectra for histidine competition (Figures S12 and S13), literature log β values for Cu(II) complexes of hepc6 and Aβ4-16 (Tables S1 and S2), species distributions for Cu(II) complexes of MNH-NH2, Aβ4-9, Aβ4-16, and MDH-NH2 (Figure S14), stopped-flow spectra (Figure S15), kinetic traces (Figure S16), and recalculation of apparent spray pH (Table S3) (PDF)
