# Supplementary material for: Electrospray-Induced Mass Spectrometry Is Not Suitable for Determination of Peptidic Cu(II) Complexes
Source: J Am Soc Mass Spectrom. 2021 Nov 5;32(12):2766–76. doi: 10.1021/jasms.1c00206 (PMC8640992; doi:10.1021/jasms.1c00206)
Supplement: Supplementary file 1 — js1c00206_si_001.pdf [file js1c00206_si_001.pdf]

SUPPORTING INFORMATION

Electrospray-induced mass spectrometry is not suitable for determination of peptidic Cu(II) complexes

Dawid Płonka, Radosław Kotuniak, Katarzyna Dąbrowska, Wojciech Bal*
Institute of Biochemistry and Biophysics, Polish Academy of Sciences,
Pawińskiego 5A, 02-105, Warsaw, Poland

Corresponding Author: * wbal@ibb.waw.pl

Supporting figures and tables

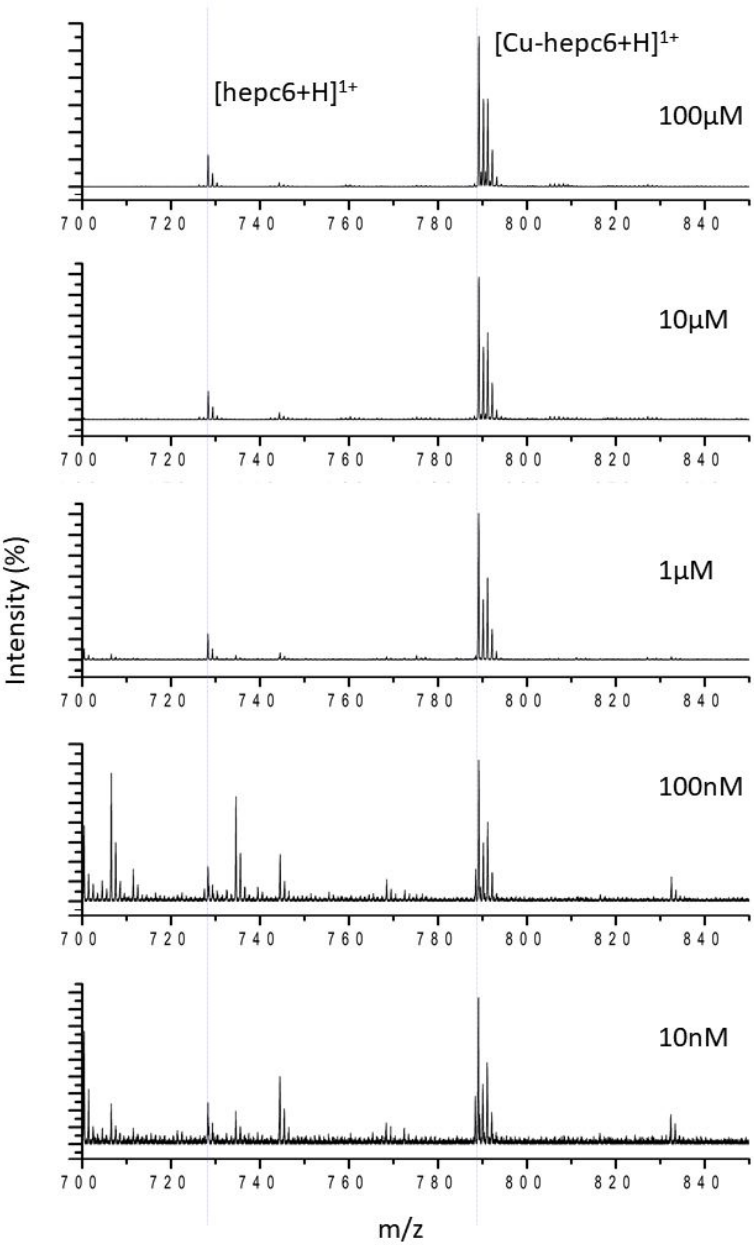

Figure S1. *The ESI-MS spectra of the serial dilution of the Cu(II)hepc6 complex (ratio 1:1, singly charged ions). Dotted lines mark monoisotopic peaks of free hepc6 and its Cu(II) complex.*

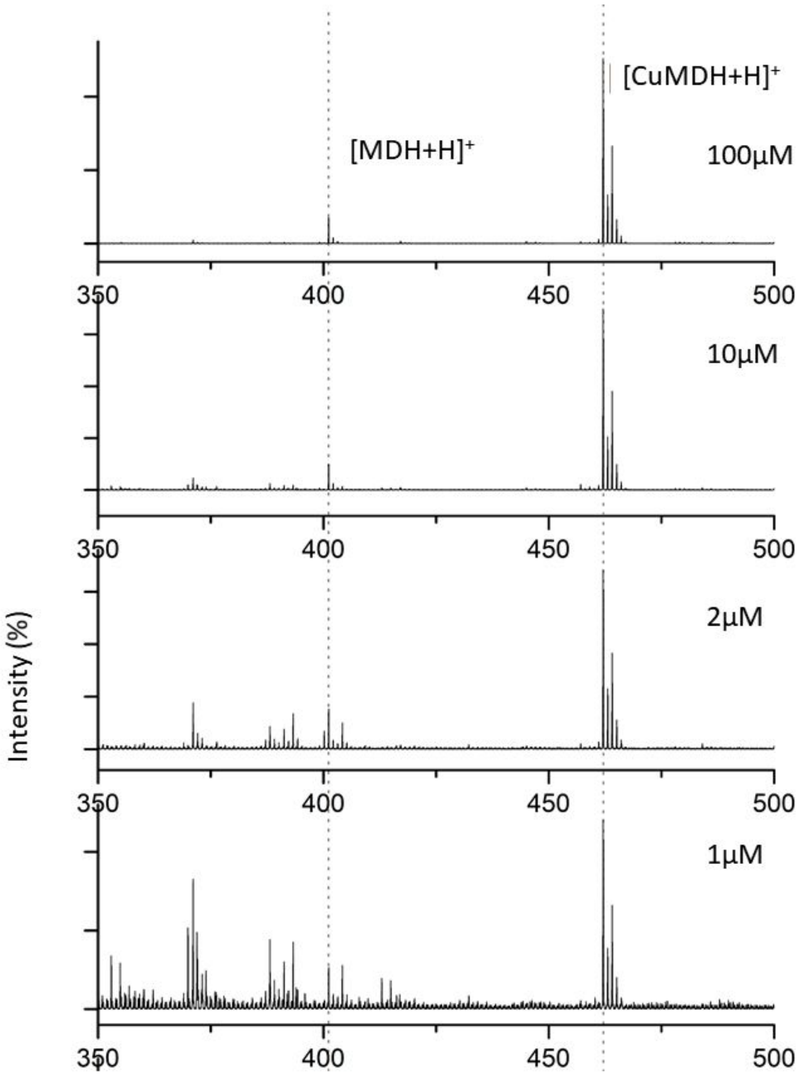

Figure S2. *The ESI-MS spectra of the serial dilution of Cu(II)MDH-NH$_2$ (ratio 1:1, singly charged ions). Dotted lines mark monoisotopic peaks of the free peptide and its Cu(II) complex.*

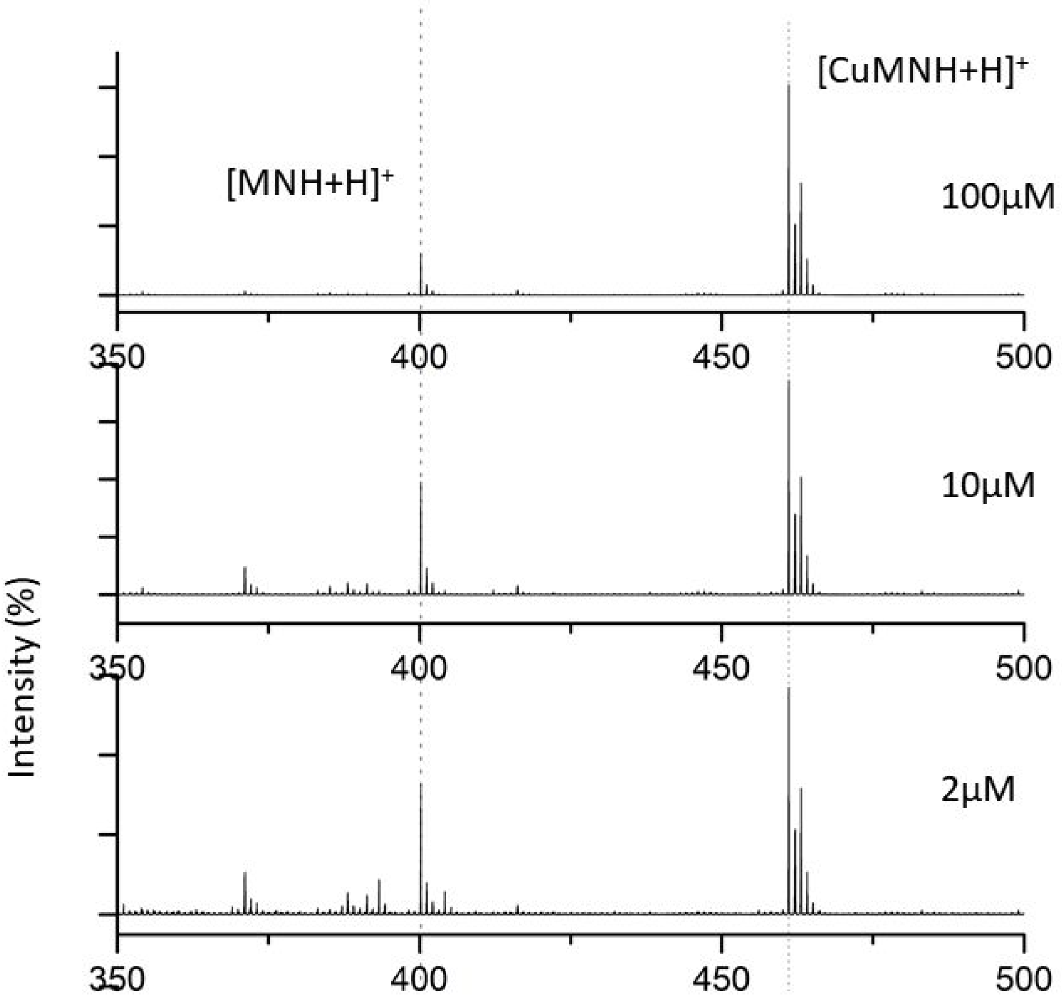

Figure S3. *The ESI-MS spectra of the serial dilution of Cu(II)MNH-NH$_2$ complex (ratio 1:1, singly charged ions). Dotted lines mark monoisotopic peaks of the free peptide and its Cu(II) complex.*

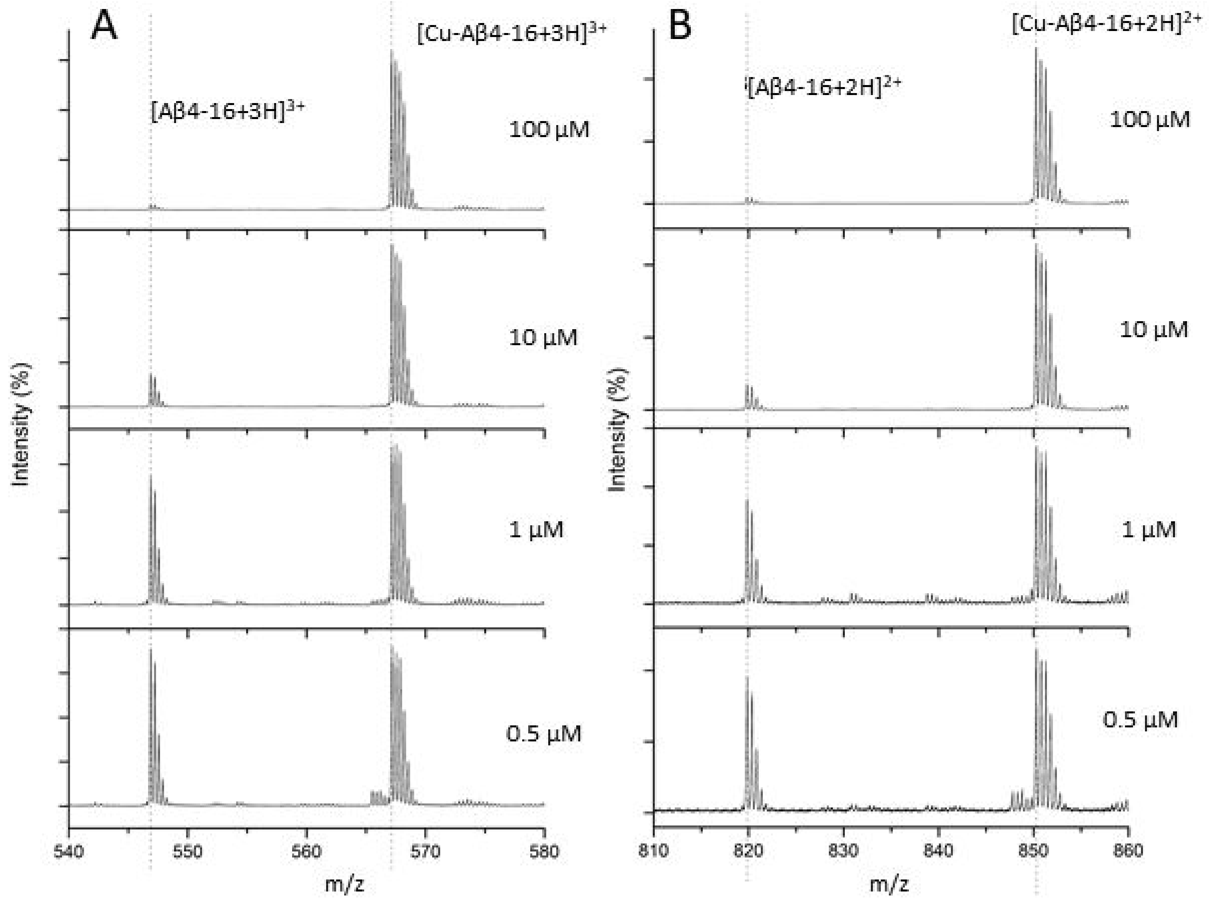

Figure S4. *The segments of ESI-MS spectra of serial dilution of the Cu(II)Aβ4-16 complex (ratio 1:1), zoomed in on triply (A) and doubly (B) charged ions. Dotted lines mark monoisotopic peaks of free peptides and their Cu(II) complexes.*

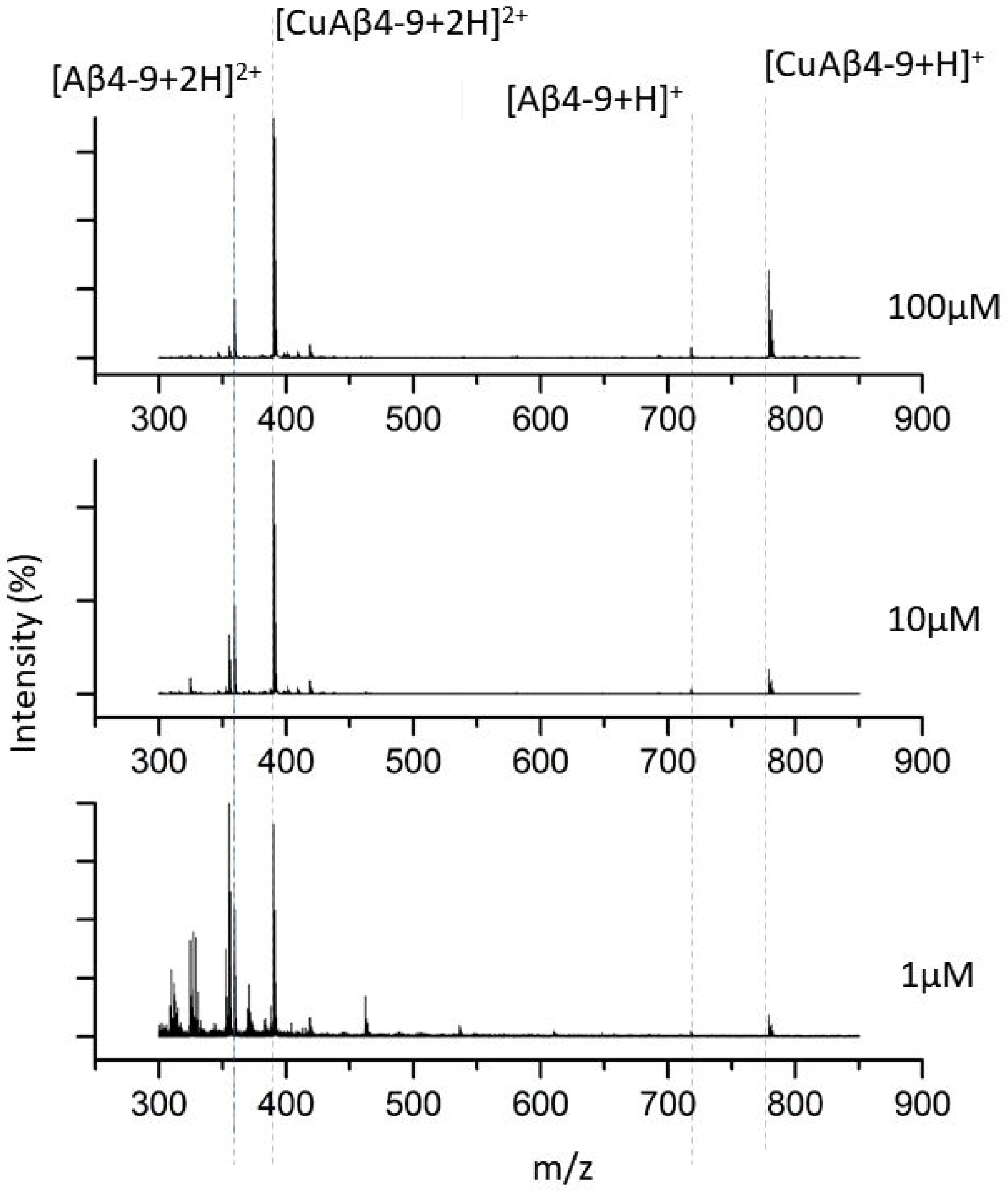

Figure S5*. The ESI-MS spectra of serial dilution of the Cu(II)Aβ4-9 complex (ratio 1:1). Dotted lines mark monoisotopic peaks of the free peptide and its Cu(II) complex.*

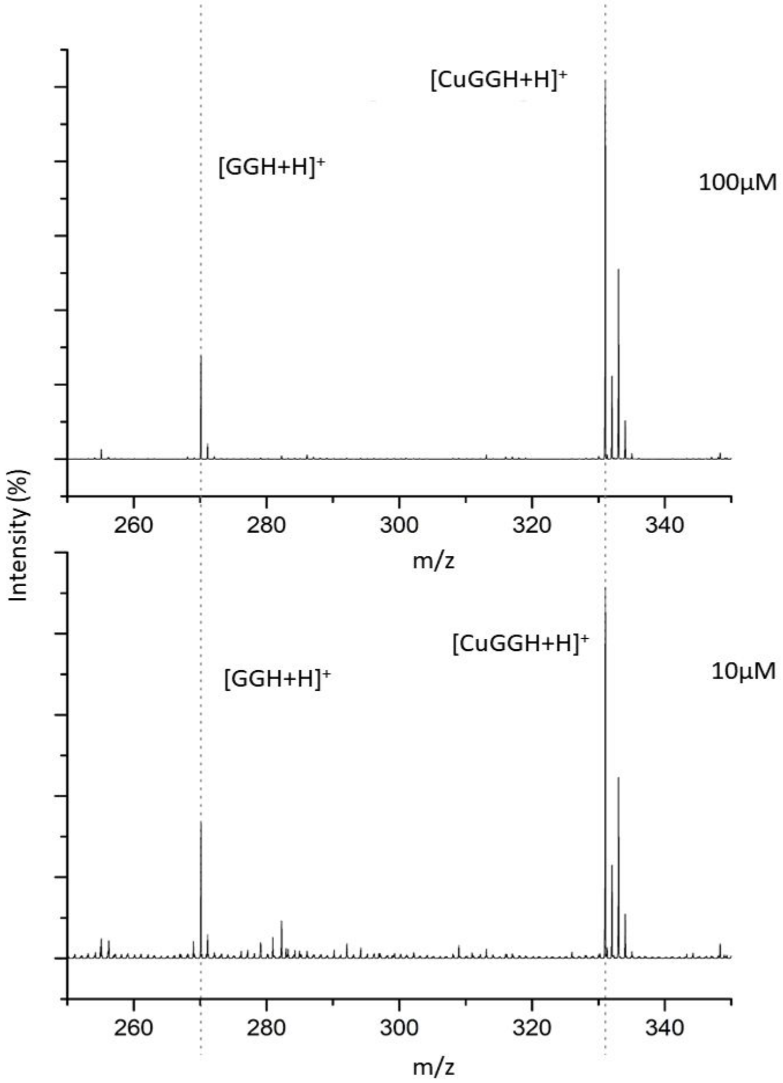

Figure S6. *The ESI-MS spectra of serial dilution of the Cu(II)GGH complex (ratio 1:1). Dotted lines mark monoisotopic peaks of the free peptide and its Cu(II) complex.*

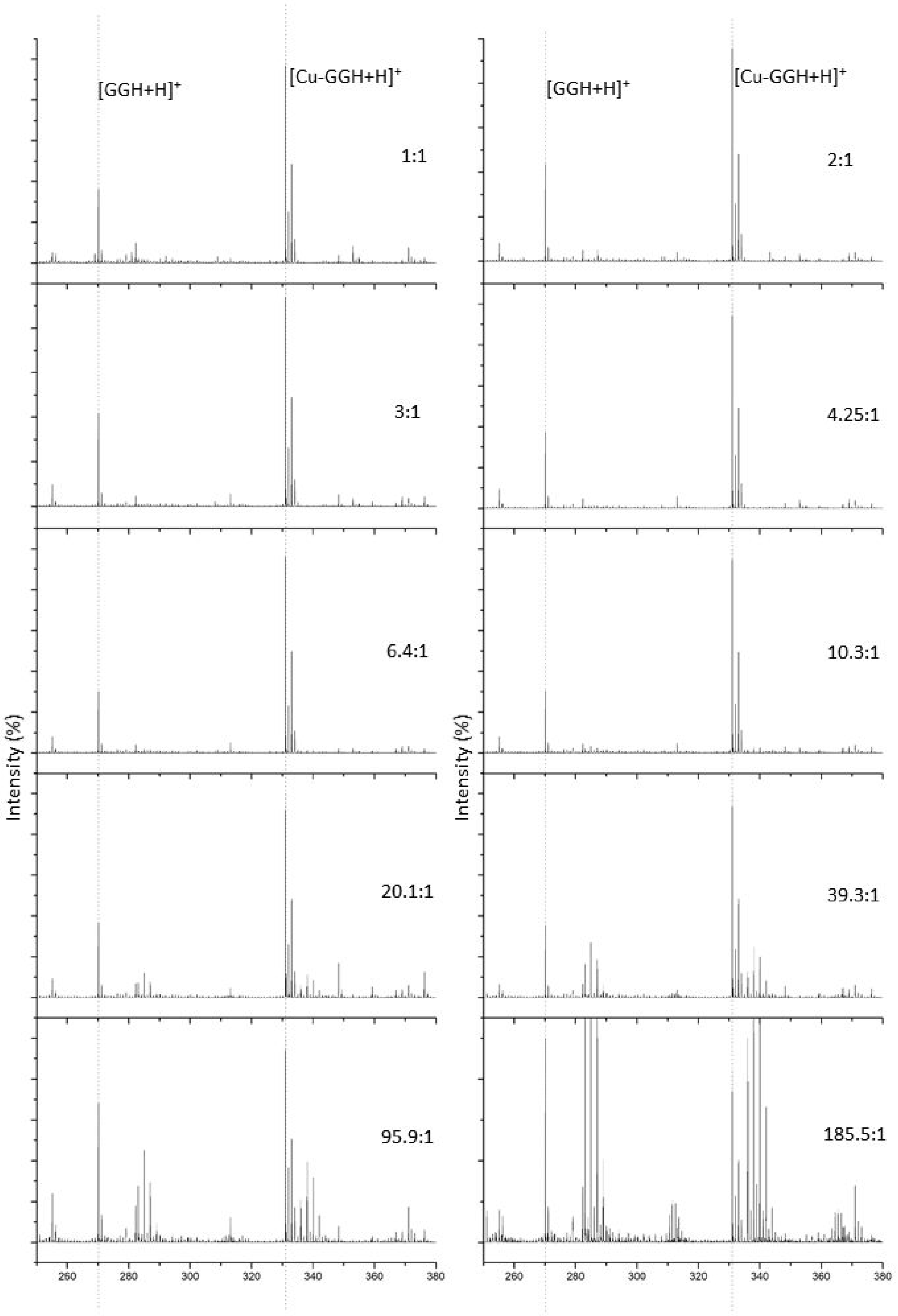

Figure S7. *Dependence of ESI-MS spectra on the Cu2+:GGH ratio, for total 10 µM GGH. The ratios are given in the spectra. Dotted lines mark monoisotopic singly charged peaks of the complex and the free peptide. Unidentified spectral impurities produced much stronger signal for 185.5:1 Cu:peptide ratio than the complex itself so that spectrum is zoomed in for better complex visibility. The manifestation of these impurities is probably due to quenching the main signals resulting from the instrument detector saturation by the Cu^{2+} ions.*

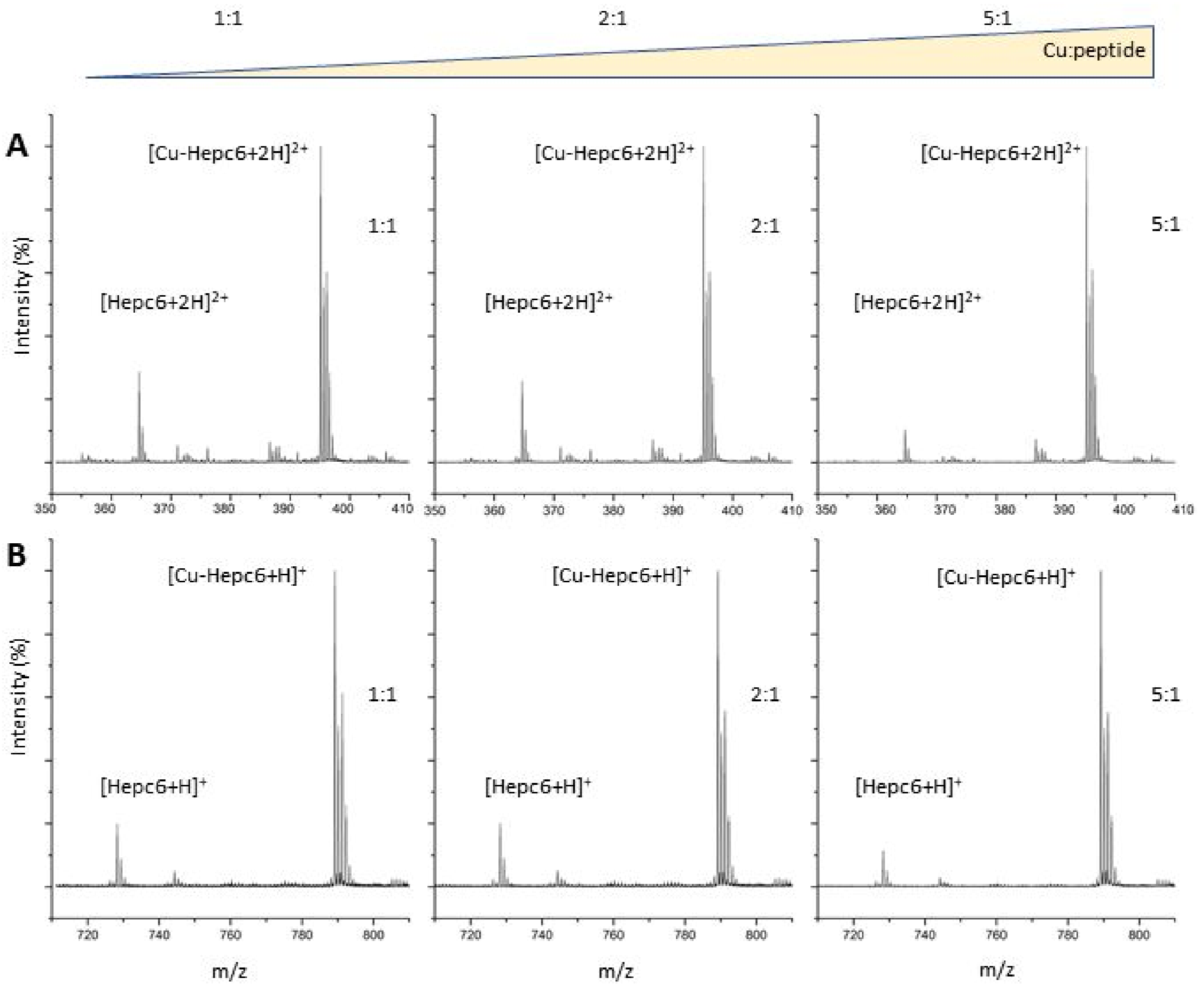

Figure S8. *Dependence of the ESI-MS spectra of doubly (A) and singly (B) charged ions on the Cu(II):hepc6 ratio, for10 µM peptide. The ratios are given in the spectra.*

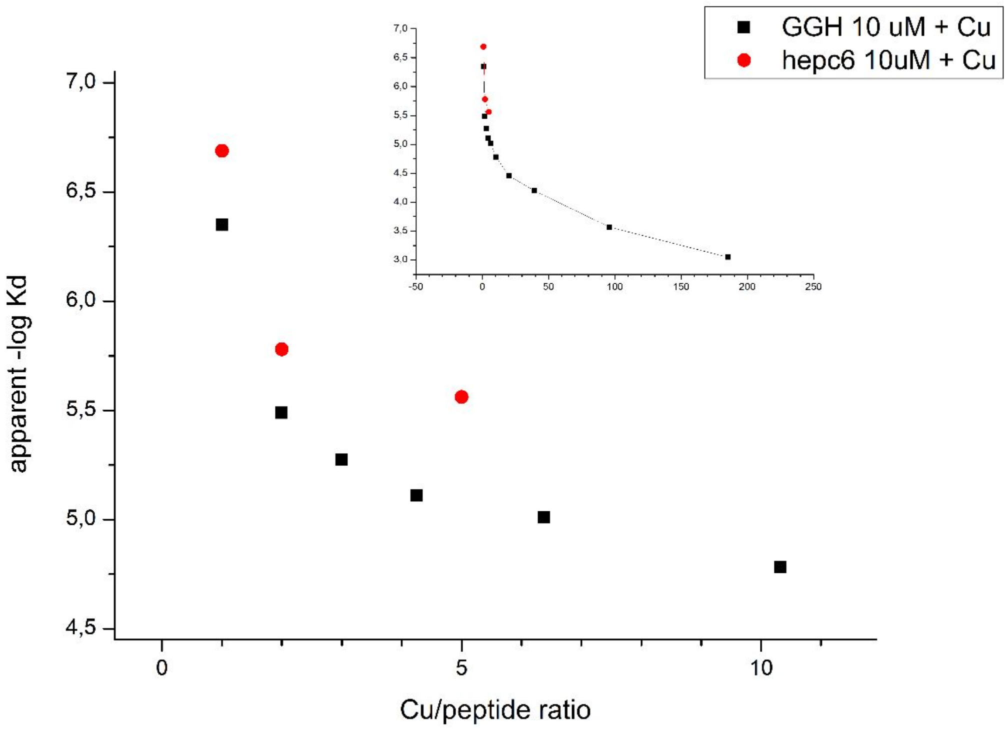

***Figure S9**. Apparent Cu(II) binding constant dependent on Cu(II)/peptide ratio from ESI-MS experiments (Fig S7 and S8) with GGH and hepc6 peptides. Inset better displays full range of Cu:GGH experiment.*

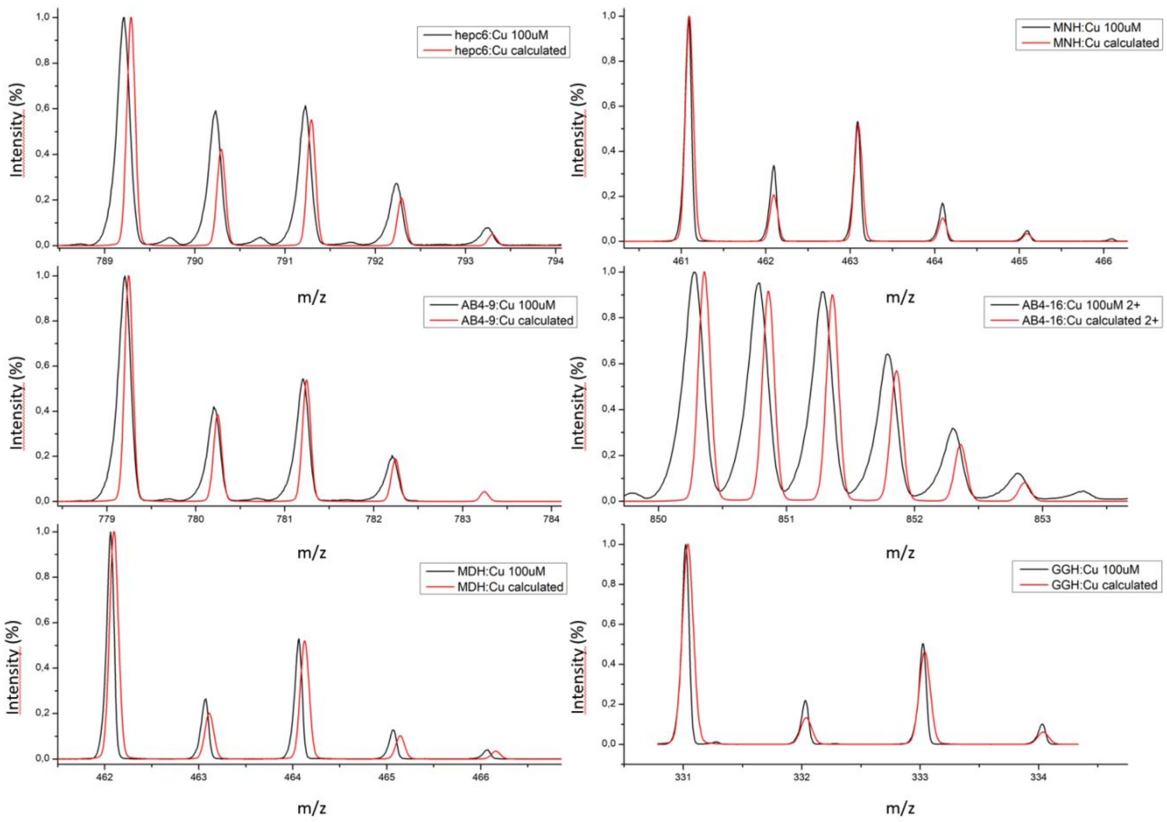

Figure S10. *Experimental and calculated isotopic distributions of 100μM peptide:Cu(II) complexes. All are singly charged ions of 100μM complexes, except for Cu:Aβ4-16 (doubly charged ions).*

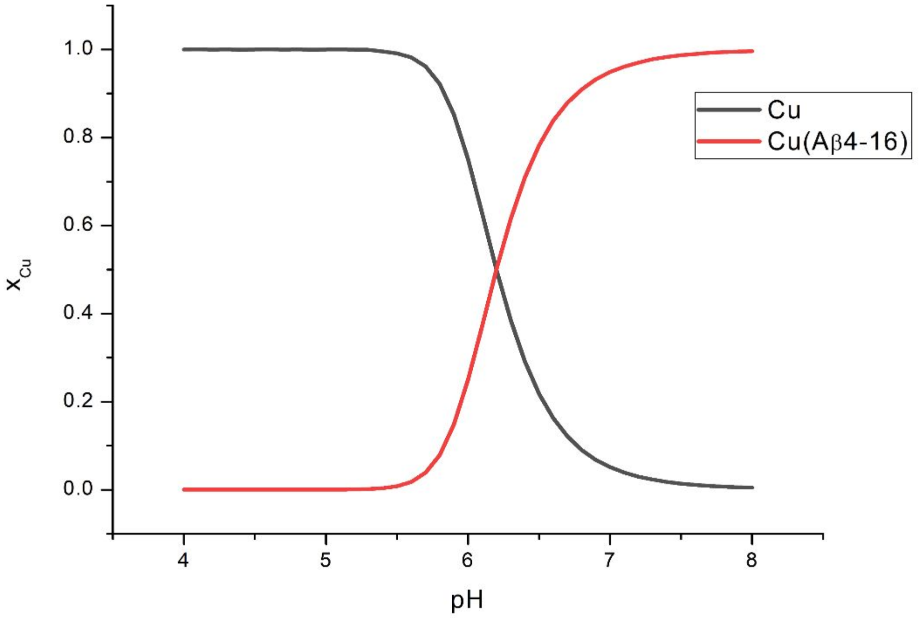

Figure S11. *The pH dependence of Cu(II) fraction bound to Aβ4-16 in a 1:1 ratio at 100 pM concentration, based on [6].*

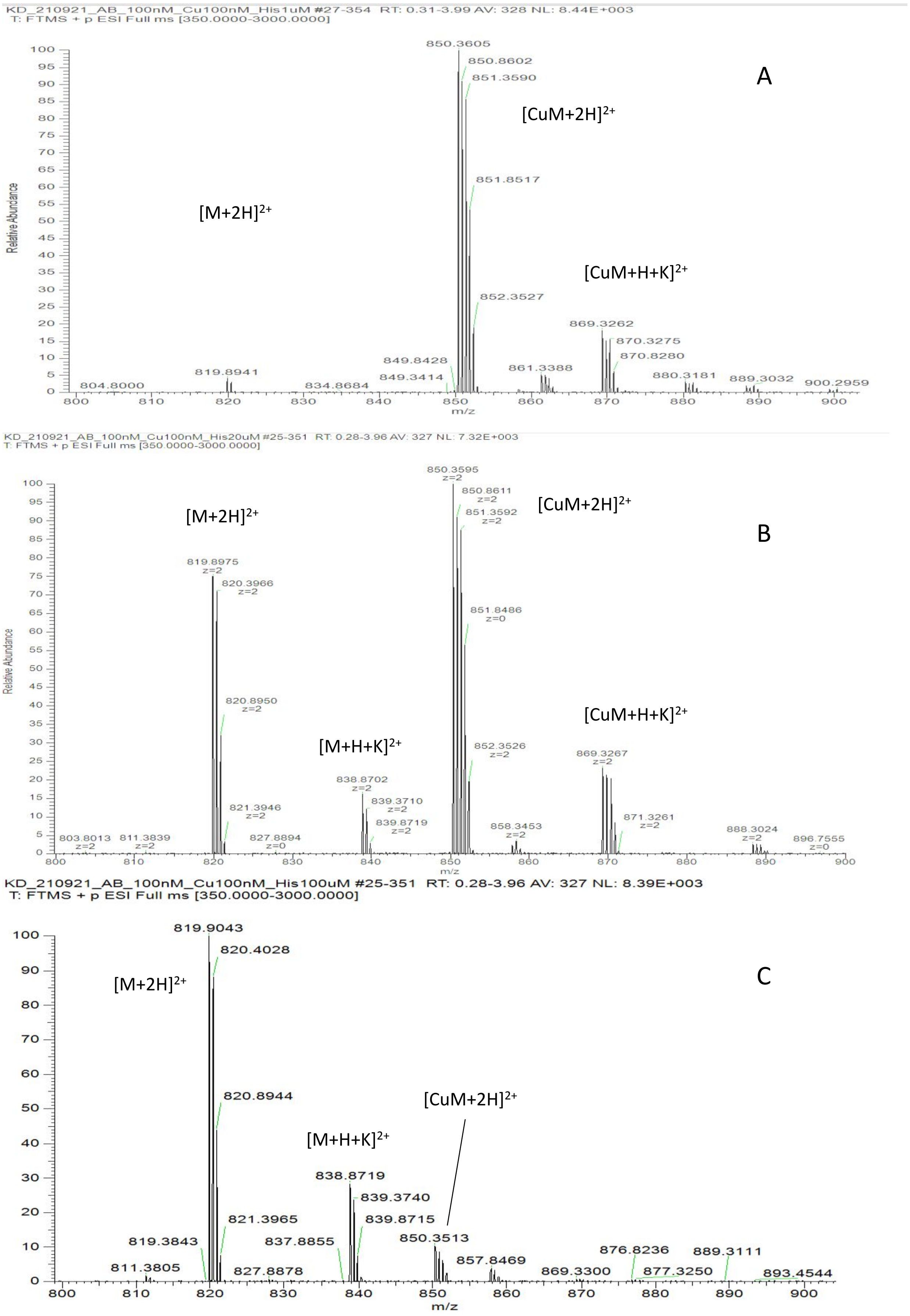

***Figure S12**. Raw spectra of 100 nM Cu(II)Aβ4-16 in competition with histidine: 1 µM (A), 20 µM (B) and 100 µM (C).*

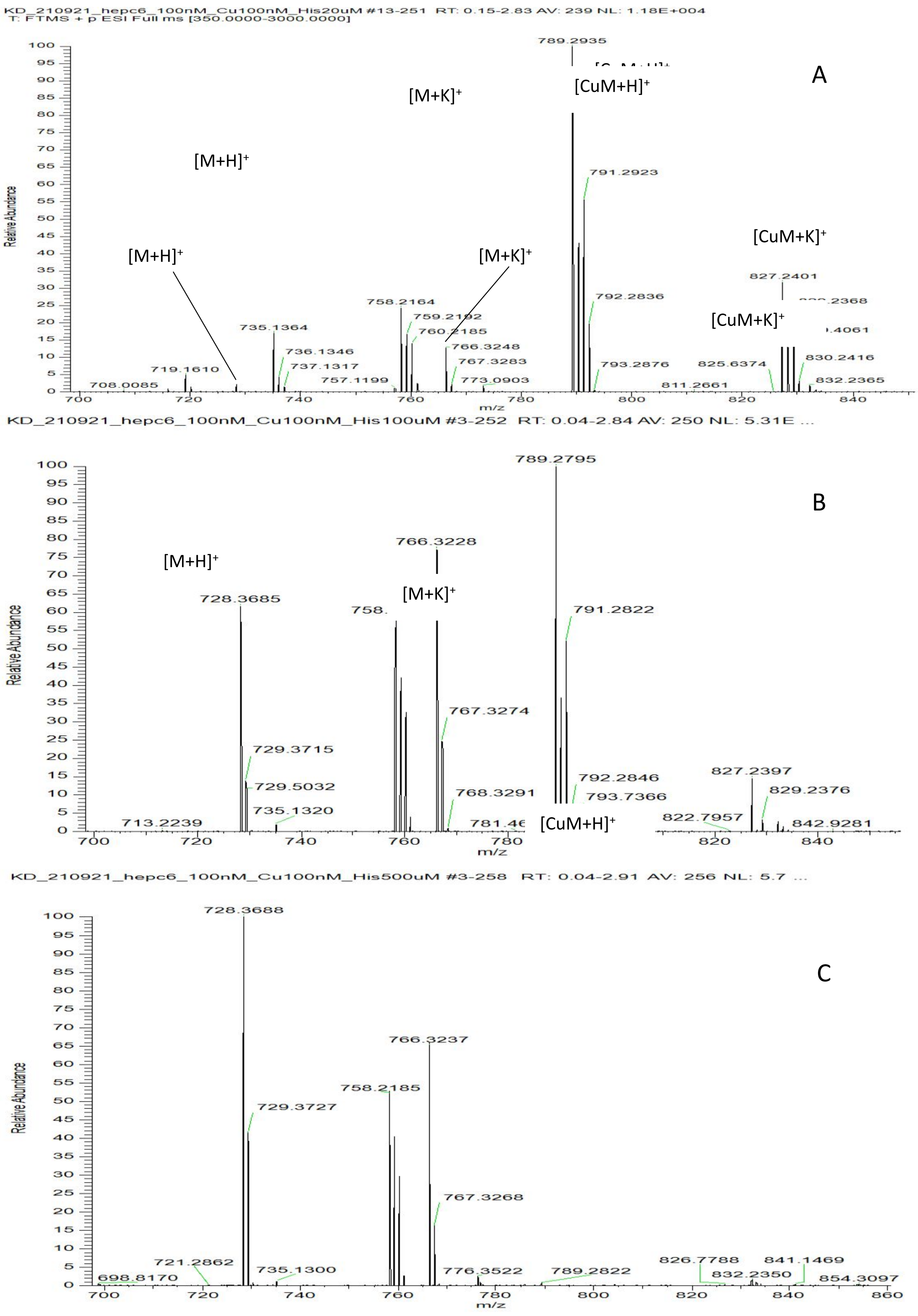

Figure S13. *Raw spectra of 100 nM Cu(II)hepc6 in competition with histidine: 20 µM (A), 100 µM (B) and 500 µM (C).*

Table S1. Protonation and stability constants (log β values) for Aβ4-16 (L) and its Cu(II) complexes and histidine (A) and its Cu(II) complexes at I = 0.1 M (KNO_3) and 25°C. Adapted from [6] and [67], respectively.

**Species**	**Log β**
H_8L	53.630
H_7L	50.651
H_6L	46.546
H_5L	40.843
H_4L	34.501
H_3L	27.801
H_2L	20.188
HL	10.297
L	
CuH_3L	37.496
CuH_2L	32.518
CuHL	26.419
CuL	19.13
$CuH_{-1}L$	9.07
$CuH_{-2}L$	-1.15
Cu_2HL	30.87
Cu_2L	25.365
$Cu_2H_{-1}L$	18.57
$Cu_2H_{-2}L$	10.29
$Cu_2H_{-3}L$	1.03
$Cu_2H_{-4}L$	-9.11
$Cu_2H_{-5}L$	-19.72
HA	9.164
H_2A	15.265
H_3A	17.35
CuHA	14.538
$CuHA_2$	24.189
CuA	10.335
CuA_2	18.307

Table S2. Protonation and stability constants (log β values) for hepc6 (L) and its Cu(II) complexes and histidine (A) and its Cu(II) complexes at I = 0.1 M (KNO_3) and 25°C. Adapted from [63] and [67], respectively.

**Species**	**Log β**
H_3L	16.99
H_2L	14.23
HL	7.77
CuHL	13.82
$CuH_{-2}L$	0.41
HA	9.164
H_2A	15.265
H_3A	17.35
CuHA	14.538
$CuHA_2$	24.189
CuA	10.335
CuA_2	18.307

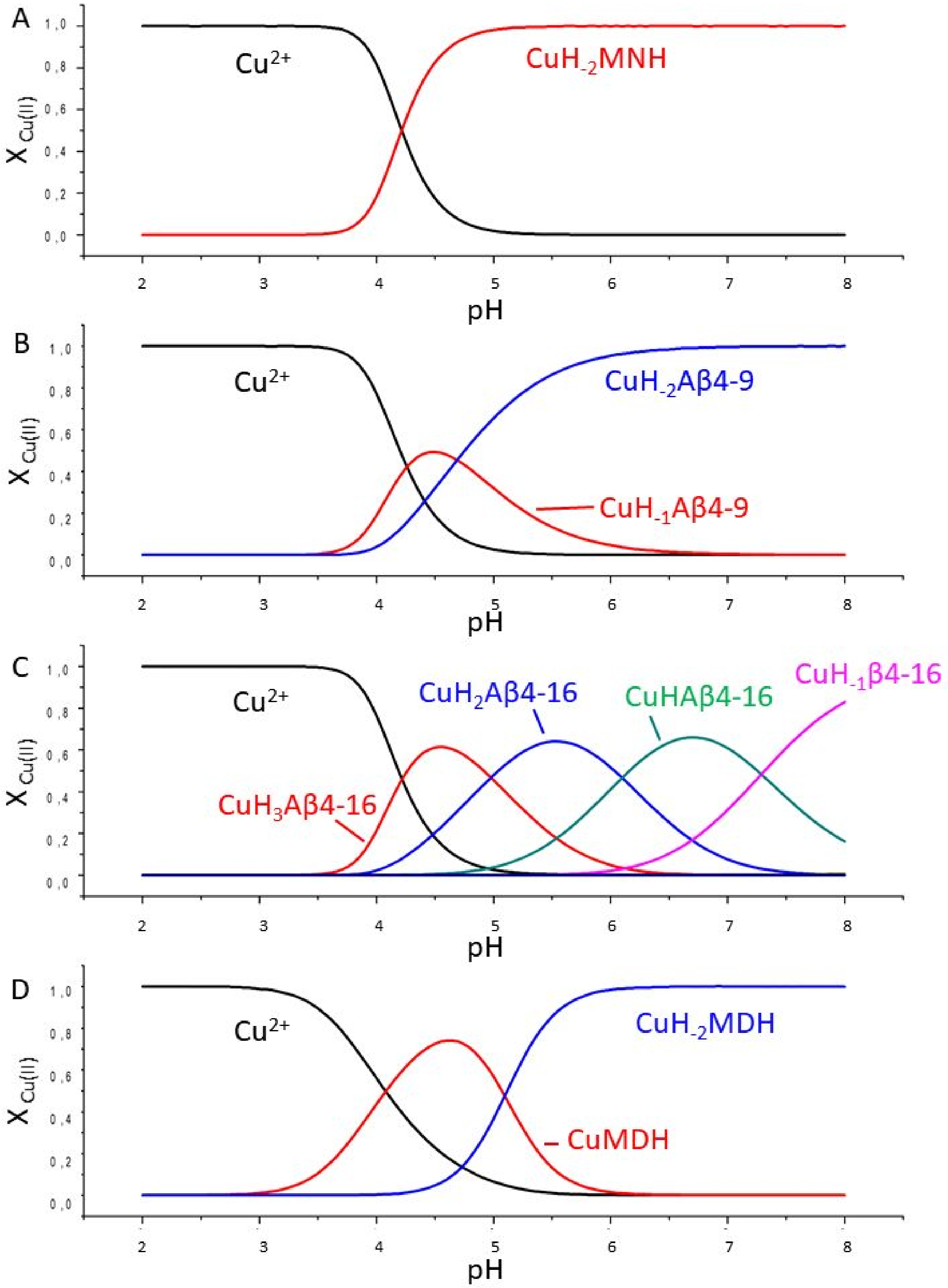

Figure S14. *The pH-dependent species distribution of Cu(II) complexes of MNH-NH$_2$ (A), Aβ4-9 (B), Aβ4-16 (C) and MDH-NH$_2$ (D), calculated for 1 mM peptides and Cu(II) recalculated from potentiometric data from [64] (A, D), [65] (B) and [6] (C).*

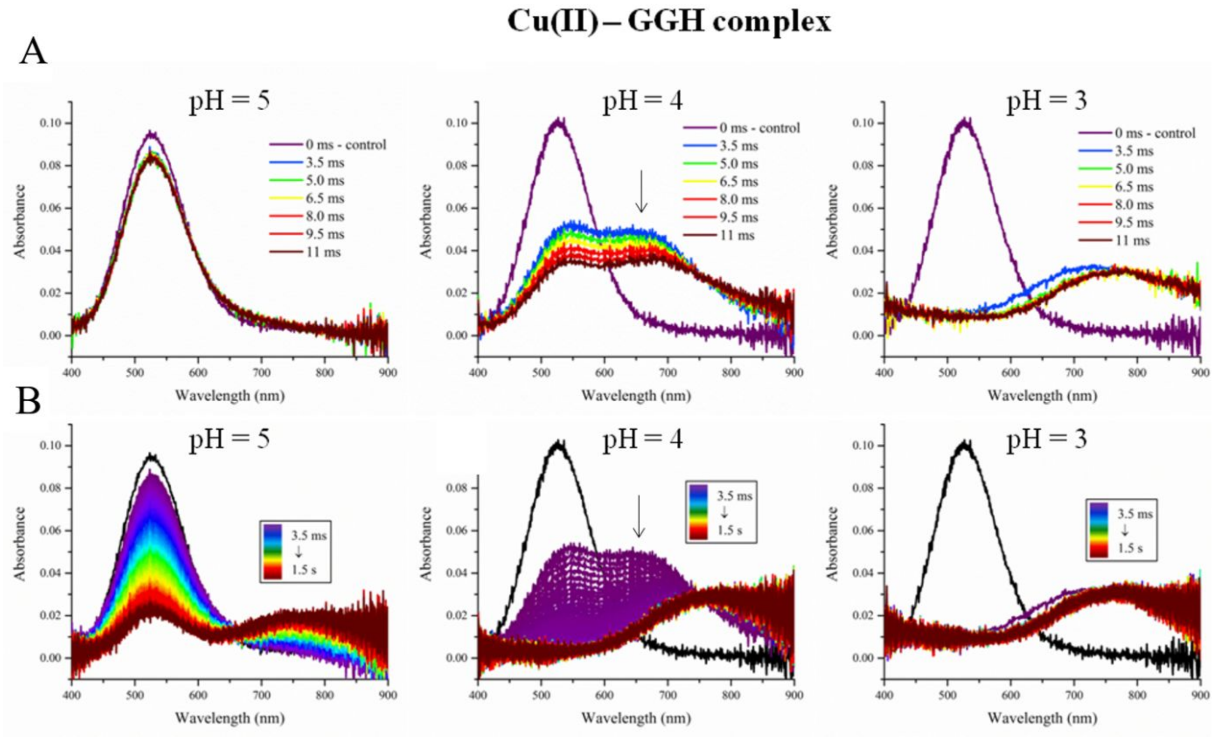

Figure S15. *Stopped-flow spectra of Cu(II)-GGH im ammonium acetate at pH=7.4 subjected to rapid pH decrease by mixing with acetic acid.* ***(A)*** *first 11 ms of the reaction,* ***(B)*** *all spectra recorded until the equilibrium was reached. Colored spectra were recorded every 1.5 ms with the instrument dead time of 2 ms. The band at 525 nm represents the 4N ATCUN complex, stable at pH=7.4. the band at 750 nm represents the intermediate 2N complex. Black arrow shows band not detectable for complex formation during pH increase.*

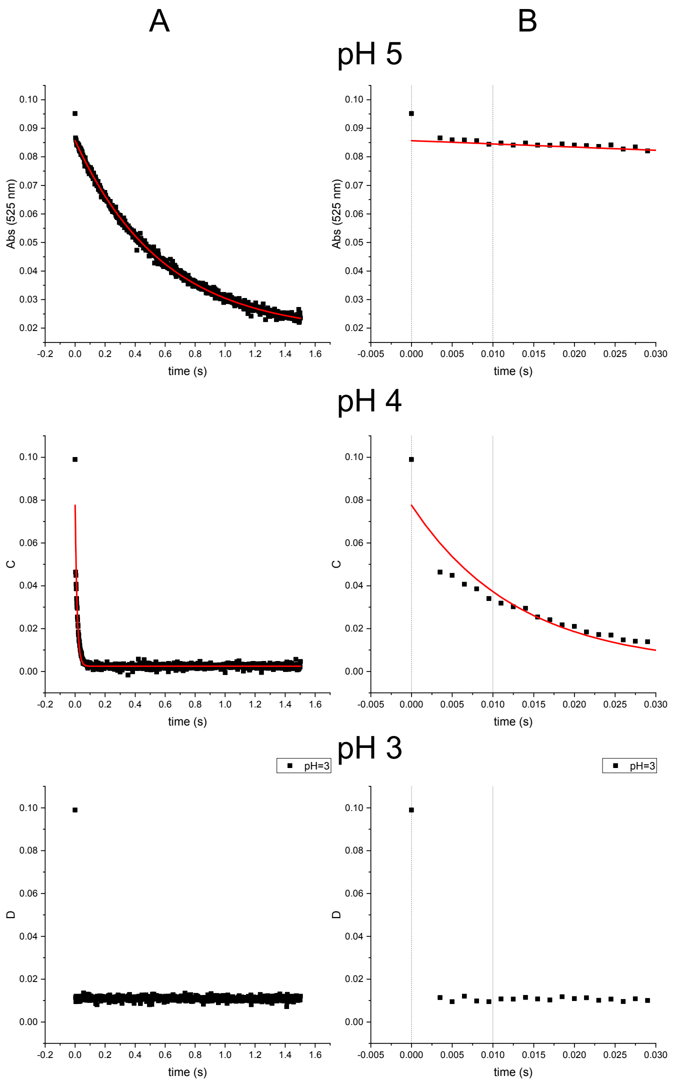

Figure S16. *Kinetic traces at 525 nm for pH-jump experiments on Cu(II)-GGH, presented in Figure S10. A. Full traces recorded over 1.5 s, and 1st order kinetic fits (red lines). B. The first 30 ms of traces presented in A. Vertical lines mark the 0 ms (reaction start) and the 10 ms time points.*

Table S3. Recalculation of apparent pH of spray droplets after inclusion of kinetic data.

A	B	C	D	E	
Hepc6	Fraction of 525 nm signal retained after 10 ms from 1st order fitting<br><br>**(X)**	signal expected at equilibrium: 4N+2N/apo (Fig. 4)<br><br>**(Y/Z)**	Complex/apo Signal expected for ESI (column C, corrected for the extent of reaction in column B)<br><br>**(1-(1-X)×Z) /((1-X)×Z)**	ESI-MS From Figs. 2 and S5, 1+ ions, 1:1 ratio (100µM Cu-peptide)	Estimated pH (comparison of columns D and E)
pH 5	0.9993	1/0	1/0	0.87/0.13	between 3 and 4
pH 4	0.9419	0.97/0.03	1/0		
pH 3	0.7432	0.18/0.82	0.79/0.21		
GGH					
pH 5	0.983	0.656/0.344	0.66/0.34	0.81/0.19	above 5
pH 4	0.4625	0.07/0.93	0.50/0.50		
pH 3	0	0/1	0/1		
